# The T cell CD6 receptor operates a multitask signalosome with opposite functions in T cell activation

**DOI:** 10.1084/jem.20201011

**Published:** 2020-10-30

**Authors:** Daiki Mori, Claude Grégoire, Guillaume Voisinne, Javier Celis-Gutierrez, Rudy Aussel, Laura Girard, Mylène Camus, Marlène Marcellin, Jérémy Argenty, Odile Burlet-Schiltz, Frédéric Fiore, Anne Gonzalez de Peredo, Marie Malissen, Romain Roncagalli, Bernard Malissen

**Affiliations:** 1 Centre d’Immunologie de Marseille-Luminy, Aix Marseille Université, Institut National de la Santé et de la Recherche Médicale, Centre National de la Recherche Scientifique, Marseille, France; 2 Centre d’Immunophénomique, Aix Marseille Université, Institut National de la Santé et de la Recherche Médicale, Centre National de la Recherche Scientifique, Marseille, France; 3 Institut de Pharmacologie et de Biologie Structurale, Université de Toulouse, Centre National de la Recherche Scientifique, Université Paul Sabatier, Toulouse, France

## Abstract

To determine the respective contribution of the LAT transmembrane adaptor and CD5 and CD6 transmembrane receptors to early TCR signal propagation, diversification, and termination, we describe a CRISPR/Cas9–based platform that uses primary mouse T cells and permits establishment of the composition of their LAT, CD5, and CD6 signalosomes in only 4 mo using quantitative mass spectrometry. We confirmed that positive and negative functions can be solely assigned to the LAT and CD5 signalosomes, respectively. In contrast, the TCR-inducible CD6 signalosome comprised both positive (SLP-76, ZAP70, VAV1) and negative (UBASH3A/STS-2) regulators of T cell activation. Moreover, CD6 associated independently of TCR engagement to proteins that support its implication in inflammatory pathologies necessitating T cell transendothelial migration. The multifaceted role of CD6 unveiled here accounts for past difficulties in classifying it as a coinhibitor or costimulator. Congruent with our identification of UBASH3A within the CD6 signalosome and the view that CD6 constitutes a promising target for autoimmune disease treatment, single-nucleotide polymorphisms associated with human autoimmune diseases have been found in the *Cd6* and *Ubash3a* genes.

## Teaser

Mori et al. established the composition of the LAT, CD5, and CD6 signalosomes of primary T cells. Positive and negative functions were solely assigned to the LAT and CD5 signalosomes, respectively, whereas the CD6 signalosome conveys antithetical functions with implications for autoimmune diseases.

## Introduction

Following TCR triggering, the LAT transmembrane adaptor assembles a multimolecular signaling complex known as the LAT signalosome (Balagopalan et al., 2010). Although the LAT signalosome ensures the propagation and diversification of TCR signals, it does not work in isolation, and other T cell surface receptors regulate early T cell activation. Among them stand CD5 and CD6, which belong to the scavenger receptor cysteine-rich superfamily and constitute paralogs that extensively diverged (Gaud et al., 2018; Padilla et al., 2000). Upon TCR-induced tyrosine phosphorylation, CD5 and CD6 assemble poorly defined signalosomes (Burgess et al., 1992; Wee et al., 1993) independently of LAT and with kinetics and in numbers comparable to those of the canonical LAT signalosome (Roncagalli et al., 2014; Voisinne et al., 2019). It thus remains to determine the composition of the LAT, CD5, and CD6 signalosomes in primary T cells and quantify their respective contributions to early TCR signal propagation and termination.

CD5 is expressed on all T cells and on a B cell subset (Brown and Lacey, 2010). On T cells, it colocalizes with the TCR at the immunological synapse (IS) and negatively regulates TCR signals in response to foreign peptides bound to MHC molecules (Azzam et al., 2001; Brossard et al., 2003; Peña-Rossi et al., 1999; Tarakhovsky et al., 1995). Although high CD5 expression levels on naive T cells have been correlated with high TCR self-reactivity, whether CD5 also limits TCR self-reactivity remains to be determined (Hogquist and Jameson, 2014). The mechanism used by CD5 to inhibit TCR signaling remains incompletely defined (Burgueño-Bucio et al., 2019). Recent data suggest that CD5 constitutes the main T cell–surface receptor capable of recruiting the E3 ubiquitin-protein ligases CBL and CBLB in response to TCR stimulation, thereby promoting ubiquitylation of colocalized signaling effectors (Voisinne et al., 2016).

CD6 is expressed on T cells and recognizes CD166 (also known as Activated Leukocyte Cell Adhesion Molecule [ALCAM]; Chappell et al., 2015) and CD318 (Enyindah-Asonye et al., 2017). The CD6–ALCAM interaction is important for IS stabilization and sustained TCR-induced cell proliferation (Meddens et al., 2018; Zimmerman et al., 2006). Upon TCR triggering, CD6 recruits the guanine nucleotide exchange factor VAV1 (Roncagalli et al., 2014), syntenin-1 (Gimferrer et al., 2005), and the adaptor proteins SLP-76 (also known as LCP2), GRAP2, and TSAD (Breuning and Brown, 2017; Hassan et al., 2006; Hem et al., 2017). Although most of these cytosolic effectors exert positive regulatory roles in T cell activation, CD6 has also been categorized as a negative regulator of T cell activation (Gonçalves et al., 2018; Oliveira et al., 2012). Mice lacking CD6 are less prone than their WT counterpart to develop experimental autoimmune encephalomyelitis (Li et al., 2017) and T cell–mediated autoimmune retinal destruction (Zhang et al., 2018), suggesting that CD6 has a net costimulatory effect in the development of several autoimmune diseases.

Using affinity purification coupled with mass spectrometry (AP-MS), it is possible to define the constellation of proteins (“the preys”) assembling around proteins (“the baits”) of the TCR-signaling network (Roncagalli et al., 2014). Combining the resulting “interactomes” with the interaction stoichiometry and cellular abundance of the interacting proteins provides quantitative parameters for systems-level understanding of TCR signal propagation and diversification (Voisinne et al., 2019). Gene-targeted mice in which T cell proteins are tagged with an affinity Twin-Strep-tag (OST; Junttila et al., 2005) permit generation of primary T cells expressing physiological levels of signalosomes that are amenable to AP-MS (Voisinne et al., 2019). Considering that it takes up to a year to obtain such mice, we describe here a faster approach that uses primary mouse T cells and permits establishment of the composition and dynamics of signalosomes of interest in 4 mo. Accordingly, we improved recent CRISPR/Cas9–based platforms for editing primary T cells (Anderson et al., 2019; Nüssing et al., 2020; Roth et al., 2018) to enable monitoring at the single-cell level that the OST tag is properly inserted in the gene of interest and to sort the low frequency of properly edited T cells before subjecting them to AP-MS analysis. We further used this “fast-track” approach to determine the composition, stoichiometry, and dynamics of the CD5, CD6, and LAT signalosomes that assemble in primary T cells following TCR engagement, and we compared such signalosomes with the transcriptional and functional outcomes resulting from TCR activation of primary T cells lacking LAT, CD5, or CD6.

## Results

### Primary mouse T cells amenable to fast-track interactomics

**Figure 1.**
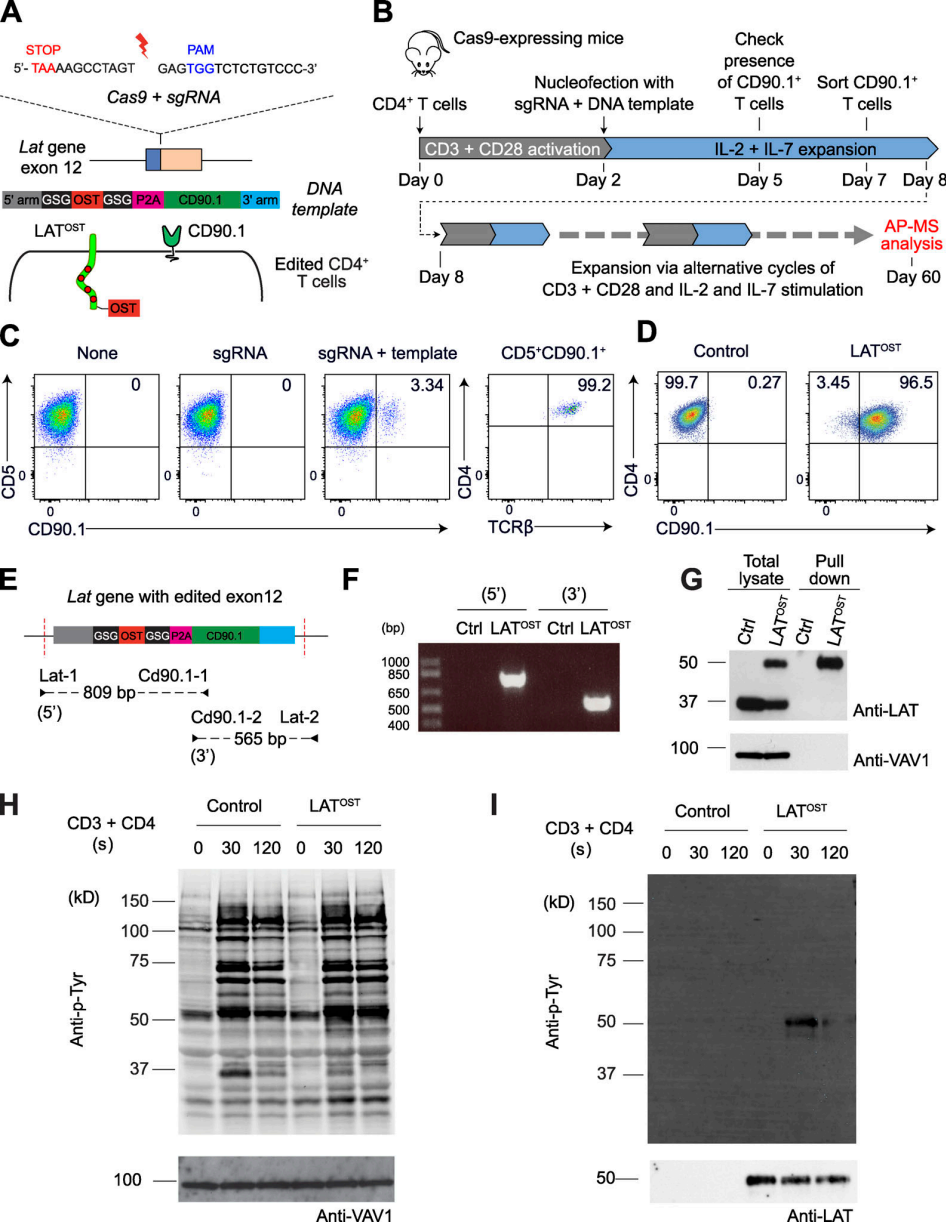
**Mouse primary CD4^+^ T cells amenable to fast-track AP-MS characterization of the LAT signalosome.** **(A)** An sgRNA was designed to introduce a double-strand break 12 bp 3′ of the stop codon found in the last exon of the *Lat* gene, and an 843-bp-long dsDNA was used as a template for HDR (see Fig. S1, A and B). Following HDR, CD4^+^ T cells are expected to coexpress LAT^OST^ and CD90.1 molecules. **(B)** Workflow used for editing, selecting, and expanding CD4^+^ T cells expressing LAT^OST^ molecules amenable to AP-MS. **(C)** T cells were analyzed for expression of CD90.1 3 d after nucleofection with vehicle alone (None), sgRNA, or sgRNA plus the HDR template (sgRNA + template). Also shown is the expression of CD4 and TCRβ on CD5^+^ CD90.1^+^ T cells. **(D)** Sorted CD90.1^+^ CD4^+^ T cells and WT CD4^+^ T cells were expanded in vitro and analyzed for expression of CD90.1 before AP-MS analysis. Data in C and D are representative of at least three experiments. **(E)** PCR genotyping schematics of sorted CD90.1^+^ CD4^+^ T cells expressing LAT^OST^ molecules. The two specified PCR primer pairs provide diagnostic bands for each junction. Correct targeting was further confirmed by sequencing the 5′ and 3′ junction fragments (Fig. S1, C and D). Also shown are the expected sizes of the PCR amplicons. **(F)** PCR genotyping was performed on WT CD4^+^ T cells (Ctrl) and on sorted CD90.1^+^ CD4^+^ T cells (LAT^OST^) using the PCR primer pairs specified in E. **(G)** Immunoblot analysis of equal amounts of proteins from WT and LAT^OST^ CD4^+^ T cell lysates that were either directly analyzed (Total lysate) or subjected to affinity purification on Strep-Tactin Sepharose beads followed by elution of proteins with D-biotin (Pull down), and both were probed with antibody to anti-LAT or anti-VAV1 (loading control). The long-term expanded LAT^OST^ CD4^+^ T cells showed an even representation of WT and LAT^OST^ alleles. **(H)** Immunoblot analysis of equal amounts of proteins from total lysates of WT and LAT^OST^ CD4^+^ T cells left unstimulated or stimulated for 30 s or 120 s with anti-CD3 and anti-CD4 and probed with antibody to phosphorylated tyrosine (Anti-p-Tyr) or anti-VAV1 (loading control). **(I)** Immunoblot analysis of equal amounts of lysates of WT and of LAT^OST^ CD4^+^ T cells stimulated as in H and subjected to affinity purification on Strep-Tactin Sepharose beads, followed by elution of proteins with D-biotin, and probed with antibody to phosphorylated tyrosine (Anti-p-Tyr) or anti-LAT. Left margin in G–I, molecular size in kilodaltons. Data in H and I are representative of at least two independent experiments. PAM, protospacer-adjacent motif. GSG, Gly-Ser-Gly spacer.

We used the *Lat* gene as a proof of concept to develop a CRISPR/Cas9–based approach permitting determination of the composition of signalosomes assembling in primary CD4^+^ T cells before and after TCR engagement. Our approach preserved cell viability while permitting monitoring at the single-cell level that the OST tag required for AP-MS analysis was properly inserted at the 3′ end of at least one allele of the targeted gene. Moreover, it allowed sorting of the low frequency of properly OST-edited T cells before subjecting them to in vitro expansion and AP-MS analysis. Accordingly, we designed a single-guide RNA (sgRNA) targeting the 3′ coding end of the *Lat* gene (Fig. 1 A and Table S1) and a 843-bp-long double-stranded DNA (dsDNA) homology-directed repair (HDR) template coding for (1) a 100-bp-long *Lat* 5′ homology arm; (2) an OST tag flanked on both sides by a Gly-Ser-Gly spacer; (3) a 19-aa-long self-cleaving peptide of the porcine teschovirus-1 2A, known as P2A (Kim et al., 2011); (4) a sequence coding for CD90.1, a protein expressed at the T cell surface; and (5) a 100-bp-long *Lat* 3′ homology arm (Fig. 1 A). Upon proper HDR, OST-tagged LAT (LAT^OST^) molecules are expressed under the control of the *Lat* gene promoter, and the P2A peptide is expected to drive stoichiometric expression of CD90.1 molecules (Fig. 1 A).

**Figure S1.**
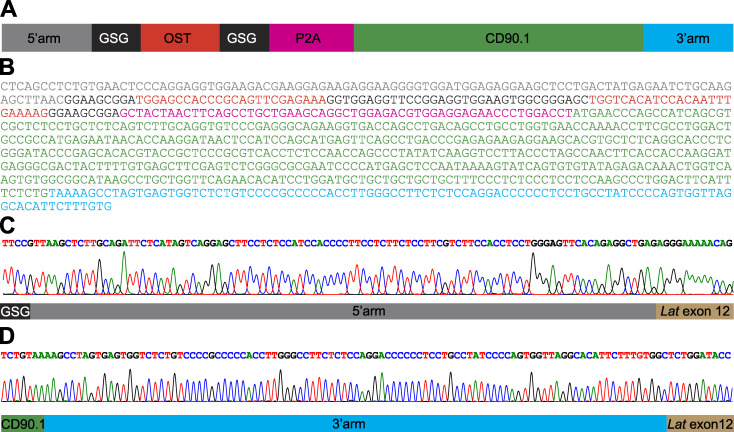
**Structure of the HDR template used to edit the *Lat* gene and sequences of the resulting junctions.** **(A)** Structure of the dsDNA HDR template used to edit the *Lat* gene. The specified sequence elements correspond to a 5′ homology arm, an OST tag flanked on both sides by a Gly-Ser-Gly spacer, a self-cleaving P2A peptide, a sequence coding for CD90.1, and a 100-bp-long 3′ homology arm. The Gly-Ser-Gly spacers are intended to give flexibility to the OST-P2A polypeptide. **(B)** Sequence of the HDR template used to edit the *Lat* gene. The encoded elements are color-coded as in A. **(C)** Sequence of the 5′ junction of the intended insertion confirmed that proper HDR occurred in the CD4^+^ T cells sorted for the expression of CD90.1. The sequencing primers correspond to Lat-1 and Cd.90.1-1 (Table S2). **(D)** Sequence of the 3′ junction of the intended insertion confirmed that proper HDR occurred in the CD4^+^ T cells sorted for the expression of CD90.1. The sequencing primers correspond to Lat-2 and Cd90.1-2 (Table S2).

Primary CD4^+^ T cells were isolated from C57BL/6 mice constitutively expressing a Cas9 endonuclease (Platt et al., 2014) and activated with anti-CD3 and anti-CD28 antibodies (Fig. 1 B). After 2 d of activation, CD4^+^ T cells were nucleofected with the sgRNA and HDR template corresponding to the *Lat* gene. C57BL/6 mice express the CD90.2 allele, permitting ready identification 4 d after nucleofection of the presence of 1.45% ± 1.69% (*n* = 5) of CD90.1^+^ T cells that retained normal TCRβ and CD4 levels (Fig. 1 C). CD90.1^+^ T cells were FACS-sorted 5 d after nucleofection (Fig. 1 D) and expanded, and their genomic DNA was analyzed by PCR using primer-pair combinations, permitting validation of the intended HDR (Fig. 1 E). Analysis of the size of the amplicons straddling the 5′ and 3′ insertion borders showed that HDR occurred correctly (Fig. 1 F), a finding corroborated via DNA sequencing (Fig. S1, C and D). Therefore, CD90.1 expression can be used as a surrogate marker permitting identification and sorting of primary CD4^+^ T cells with a properly inserted OST tag.

### Edited primary CD4^+^ T cells express LAT molecules suitable for AP-MS

The sorted CD90.1^+^ CD4^+^ T cells were expanded for approximately 2 mo to reach substantial cell numbers required for AP-MS and were subsequently denoted as long-term–expanded T cells. WT CD4^+^ T cells were expanded in parallel and used as a control. Immunoblot analysis showed that WT and CD90.1^+^ OST-edited CD4^+^ T cells expressed comparable levels of LAT and that the addition of the OST sequence resulted, as expected, in LAT^OST^ molecules with a higher molecular weight than that of WT CD4^+^ T cells (Fig. 1 G). We analyzed next whether the LAT^OST^ molecules expressed by the expanded CD90.1^+^ CD4^+^ T cells were amenable to AP-MS. After stimulation with anti-CD3 plus anti-CD4 for 30 and 120 s, the modification introduced in the *Lat* gene was without measurable effect on the global pattern of TCR-induced tyrosine phosphorylation (Fig. 1 H). LAT^OST^ bait proteins were efficiently purified using Sepharose beads coupled to Strep-Tactin (Fig. 1 I, lower panel) and underwent TCR-induced tyrosine phosphorylation that peaked 30 s after stimulation (Fig. 1 I, upper panel). As expected, no detectable material was recovered from WT CD4^+^ T cells. Therefore, upon edition of their genome and expansion in vitro, primary LAT^OST^ CD4^+^ T cells can be used for AP-MS analysis of the LAT signalosome.

### The LAT interactome of CRISPR/Cas9–edited and long-term–expanded primary CD4^+^ T cells

Long-term–expanded LAT^OST^-expressing CD4^+^ T cells were lysed with the nonionic detergent n-dodecyl-β-maltoside before or after cross-linkage of the TCR and CD4 molecules for 30 and 120 s. Protein complexes assembling around the LAT^OST^ bait were purified using Strep-Tactin Sepharose beads. After eluting samples with D-biotin, the released protein complexes were analyzed by liquid chromatography (LC) coupled to tandem mass spectrometry (MS; see Materials and methods). For each time point, three biological replicates were analyzed. To distinguish truly interacting proteins from nonspecific contaminants, we compared our data with those of control AP-MS experiments involving WT CD4^+^ T cells that went through the same in vitro expansion protocol as LAT^OST^-expressing CD4^+^ T cells.

**Figure 2.**
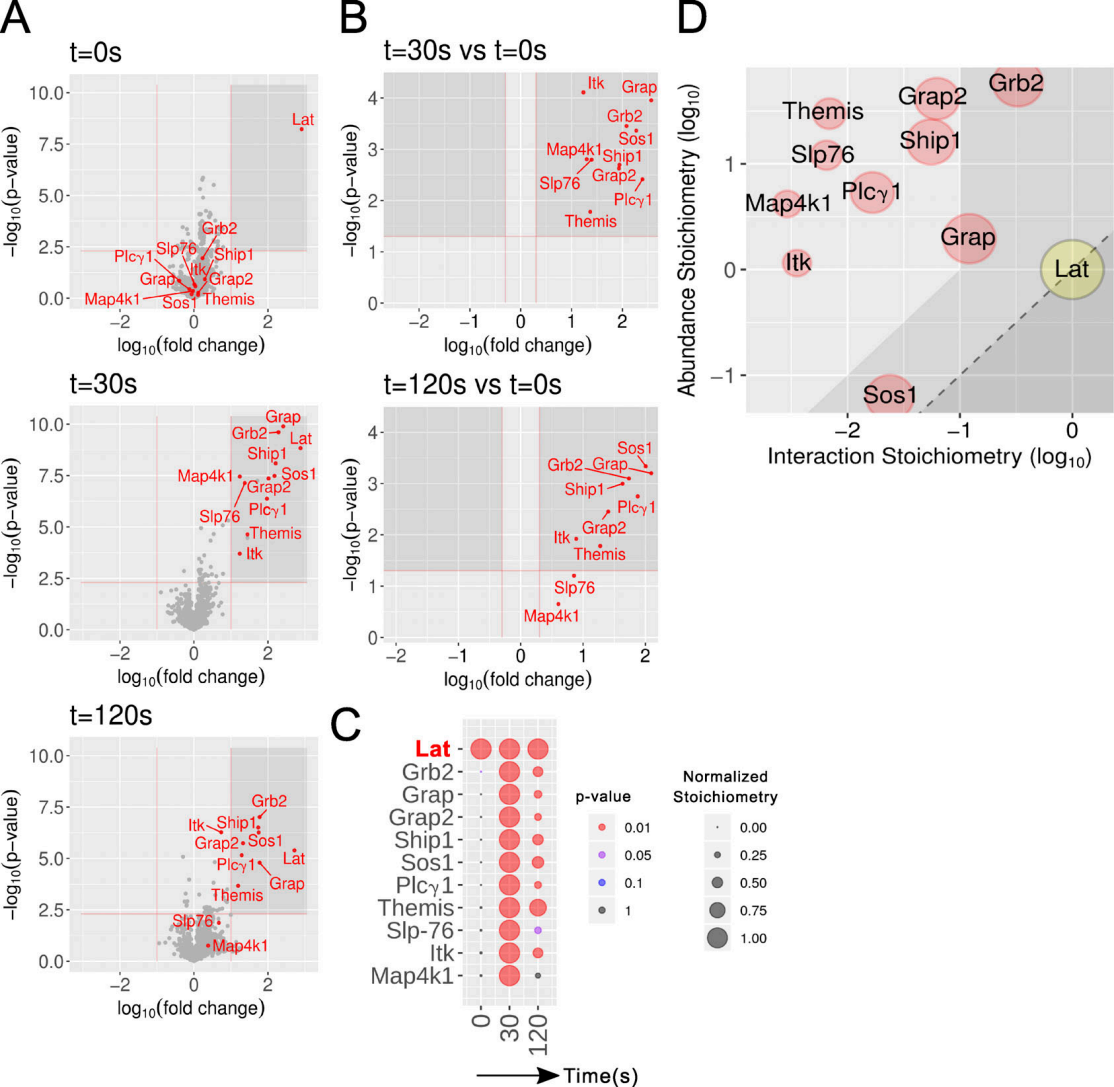
**Composition and dynamics of the LAT signalosome of long-term–expanded LAT^OST^-expressing CD4^+^ T cells.** **(A)** Volcano plot showing proteins significantly enriched after affinity purification in CD4^+^ T cells expressing LAT^OST^ molecules compared with affinity purification in control CD4^+^ T cells expressing similar levels of WT (untagged) LAT proteins before (t_0s_) and at 30 s (t_30s_) and 120 s (t_120s_) after TCR plus CD4 stimulation. **(B)** Volcano plot showing proteins significantly enriched after affinity purification in LAT^OST^-expressing CD4^+^ T cells 30 and 120 s after TCR engagement compared with affinity purification in unstimulated LAT^OST^-expressing CD4^+^ T cells. In A and B, the SLP-76, GRB2, GRAP, GRAP2, THEMIS, ITK, PLC-γ1, SHIP1, SOS1, and MAP4K1 proteins are shown in red, and the x and y axes show the average fold change (in log_10_ scale) in protein abundance and the statistical significance, respectively. **(C)** Dot plot showing the interaction stoichiometry of LAT with its 10 high-confidence preys over the course of TCR stimulation. For each LAT–prey interaction, the interaction stoichiometry has been row-normalized to its maximum value observed over the course of TCR stimulation (see normalized stoichiometry key). The 10 preys showed maximal binding to LAT after 30 s of activation. Also shown is the P value of the specified interactions (see P value key). **(D)** Stoichiometry plot of the LAT interactome. The LAT bait is specified by a yellow dot, and its 10 preys are shown as red dots. For each of these LAT–prey interactions, the ratio of prey to bait cellular abundance (“abundance stoichiometry” in log_10_ scale) was plotted as a function of the maximal interaction stoichiometry reached by the considered LAT-prey interaction over the course of TCR stimulation (“interaction stoichiometry” in log_10_ scale). For instance, LAT (41,443 copies per T cell; column G of the LAT tab in Data S2) is more abundant than SOS1 (2,562 copies per T cell), giving a ratio of prey to bait cellular abundance of −2.2 in log_10_ scale. Moreover, the maximum stoichiometry of the LAT–SOS1 interaction is reached at t_30s_ and corresponds to 0.023 (−1.6 in log_10_ scale; column D of the LAT tab in Data S2). Therefore, 953 (41,443 × 0.023) molecules of LAT are complexed to SOS1 at t_30s._ As a result, 37% (953/2,562 × 100) of the available SOS1 proteins are complexed to LAT 30 s after TCR engagement. The area including the LAT–prey interaction involving >10% of the available prey molecules is indicated in light gray and includes SOS1, GRB2, and GRAP. The limit imposed on interaction stoichiometries by the relative LAT–prey cellular abundances is shown by a dashed diagonal line that delimits a “forbidden” area (dark gray). Prey dot size is commensurate to its maximal protein enrichment over the course of stimulation.

**Figure S2.**
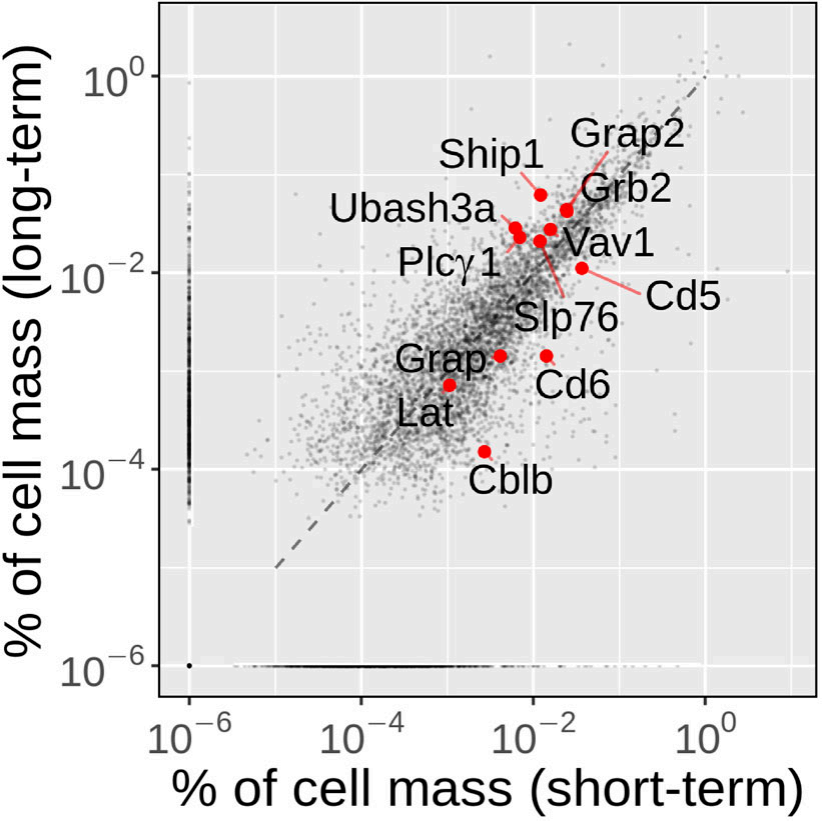
**Comparison of the fraction of cell mass occupied by the proteins quantified in both short-term– and long-term–expanded CD4^+^ T cells.** Using the “proteomic ruler” method, which relies on the MS signal of histones as an internal standard (Wiśniewski et al., 2014), we were able to quantify protein abundance for 5,773 protein groups in long-term–expanded CD4^+^ T cells (Materials and methods and Data S1), among which 5,045 could be mapped to protein groups previously quantified in short-term–expanded CD4^+^ T cells (Voisinne et al., 2019). Long-term–expanded CD4^+^ T cells had an increased cell mass (238 pg) compared with that (37 pg) of short-term–expanded CD4^+^ T cells, resulting in copy numbers per T cell generally higher in long-term–expanded CD4^+^ T cells. Comparing the fraction of cell mass corresponding to each protein present in both proteomes—a value reflecting their respective cellular concentration—showed that the fraction of cell mass corresponding to 79% of the proteins quantified in both interactomes differed by less than fourfold. However, as previously reported (Howden et al., 2019), long-term CD4^+^ T cell expansion does not scale up evenly all proteins. For instance, among the proteins relevant to the present study, some occupied a greater fraction of cell mass in long-term– than in short-term–expanded CD4^+^ T cells (CBL: 1.7-fold; ZAP70: 2.5-fold, UBASH3A: 4.7-fold), whereas other showed a converse pattern (CD5: 3.3-fold, CD6 9.8-fold, CBLB: 17.7-fold). CBLB loss has been associated with a reduced requirement for CD28 costimulation during TCR-induced cell proliferation (Bachmaier et al., 2000), and its decrease in long-term–expanded CD4^+^ T cells might confer to them a selective advantage during long-term in vitro expansion. Some of the proteins discussed in the Results are highlighted in red.

To identify the preys associating with LAT in a TCR-inducible manner, we proceeded in two steps. In the first step, we identified 10 high-confidence preys that showed a >10-fold enrichment with a P value below 0.005 in at least one of the three conditions of stimulation (0, 30, and 120 s; Fig. 2 A). In the second step, we identified among the 10 high-confidence LAT preys those whose interaction stoichiometry changed at least twofold with a P value below 0.05 following TCR plus CD4 stimulation, compared with the nonstimulated condition (Fig. 2 B). All 10 preys passed this second step and corresponded to the cytosolic adaptors SLP-76, GRB2, GRAP, GRAP2, and THEMIS, the protein tyrosine kinase (PTK) ITK, the phospholipase PLC-γ1, the phosphatidylinositol 3,4,5-trisphosphate (PI(3,4,5)P_3_) 5-phosphatase 1 SHIP1, the guanine nucleotide exchange factor SOS1, and the serine–threonine protein kinase MAP4K1 (HPK1; Fig. 2 B). The 10 TCR-inducible preys showed a transient pattern of binding to LAT that peaked 30 s after stimulation (Fig. 2 C). By combining the cellular abundances of the protein expressed in long-term–expanded CD4^+^ T cells (Fig. S2 and Data S1) and interaction stoichiometries (Data S2), the LAT signalosome was organized into a “stoichiometry plot” (Fig. 2 D; Voisinne et al., 2019). Accordingly, for each documented LAT–prey interaction, the ratio of bait to prey cellular abundance was plotted as a function of the maximal interaction stoichiometry reached by the considered bait–prey interaction over the course of TCR stimulation (Fig. 2 D). It showed, for instance, that 37% of the pool of SOS1 proteins available in CD4^+^ T cells was mobilized to interact with LAT 30 s after TCR engagement. Therefore, our approach identified in 4 mo the quantitative composition and dynamics of the LAT signalosome of primary T cells before and following TCR engagement.

### The CD6 interactome of CRISPR/Cas9–edited and long-term–expanded primary CD4^+^ T cells

**Figure S3.**
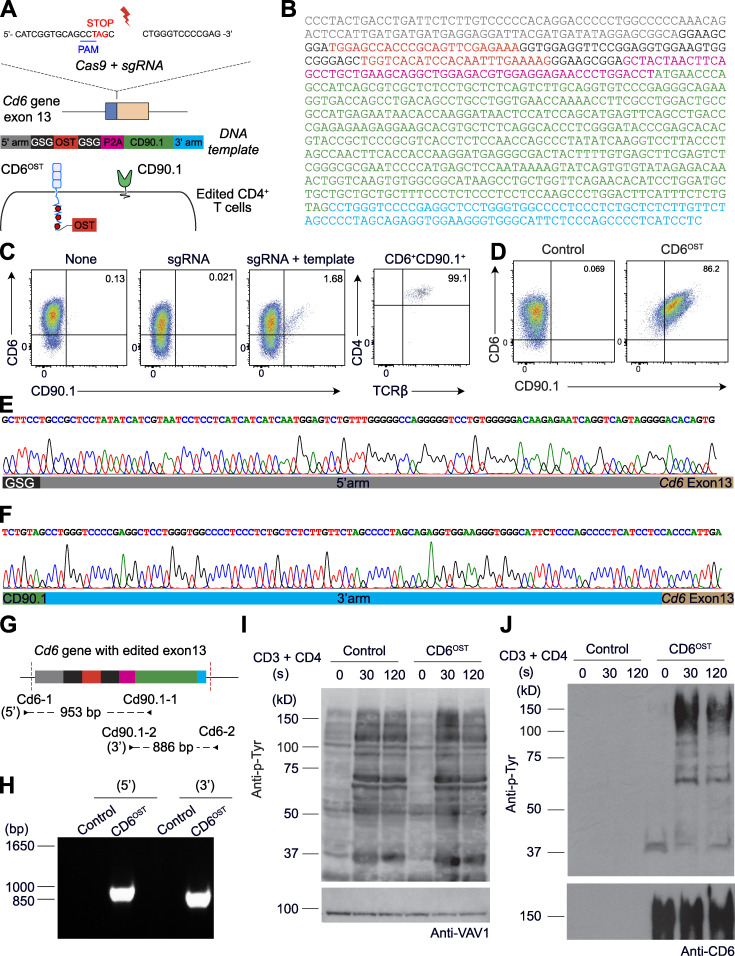
**Mouse primary CD4^+^ T cells amenable to fast-track AP-MS characterization of the CD6 signalosome.** **(A)** An sgRNA was designed to introduce a double-strand break (DSB) in the last *Cd6* exon 4 bp 5′ from the first nucleotide of the STOP codon, and an 843-bp-long dsDNA template was used for HDR. Following proper HDR, CD4^+^ T cells are expected to coexpress CD6-OST and CD90.1 molecules at their surface. **(B)** Sequence of the dsDNA HDR template used to edit the *Cd6* gene. **(C)** CD4^+^ T cells were analyzed for expression of CD6 and CD90.1 3 d after nucleofection with vehicle alone (None), sgRNA, or sgRNA plus HDR template (sgRNA + template). Note that in the absence of an HDR template, inappropriate sealing of the DSB resulted in part in the loss of the *Cd6* open reading frame and in a corresponding decrease in CD6 mean fluorescence intensity. Also shown is the expression of CD4 and TCRβ on CD6^+^CD90.1^+^ CD4^+^ T cells. The percentage of CD4^+^TCRβ^+^cells is indicated. **(D)** Sorted CD90.1^+^ CD4^+^ T cells expressing CD6^OST^ molecules (CD6^OST^) and WT (Control) CD4^+^ T cells were expanded in vitro and analyzed for expression of CD6 and CD90.1 before AP-MS analysis. In C and D, data are representative of at least three experiments. **(E)** Sequences of the 5′ junction corresponding to the intended insertion confirmed that proper HDR occurred in the primary CD4^+^ T cells sorted for the expression of CD90.1. The sequencing primers correspond to Cd90.1-1 and Cd6-1 (Table S2). **(F)** Sequences of the 3′ junction corresponding to the intended insertion confirmed that proper HDR occurred in the primary CD4^+^ T cells sorted for the expression of CD90.1. The sequencing primers correspond to Cd6-2 and Cd90.1-2 (Table S2). **(G)** PCR genotyping schematics of sorted CD90.1^+^ CD4^+^ T cells expressing CD6^OST^ molecules. The two specified PCR primer pairs provide diagnostic bands for each junction. Also shown are the expected sizes of the PCR amplicons. **(H)** PCR genotyping was performed on WT CD4^+^ T cells (Control) and on sorted CD90.1^+^ CD4^+^ T cells (CD6^OST^) using the PCR primer pairs specified in G. **(I)** Immunoblot analysis of equal amounts of proteins from total lysates of WT (Control) and CD6^OST^ CD4^+^ T cells left unstimulated or stimulated for 30 or 120 s with anti-CD3 and anti-CD4, probed with antibody to phosphorylated tyrosine (Anti-p-Tyr) and anti-VAV1 (loading control). Left margin, molecular size in kilodaltons. **(J)** Immunoblot analysis of equal amounts of lysates of WT (Control) and of CD6^OST^ CD4^+^ T cells stimulated as in I, subjected to affinity purification on Strep-Tactin Sepharose beads followed by elution of proteins with D-biotin, and probed with antibody to phosphorylated tyrosine (Anti-p-Tyr) or anti-CD6 (affinity purification control). Data in I and J are representative of at least two independent experiments. PAM, protospacer-adjacent motif.

**Figure 3.**
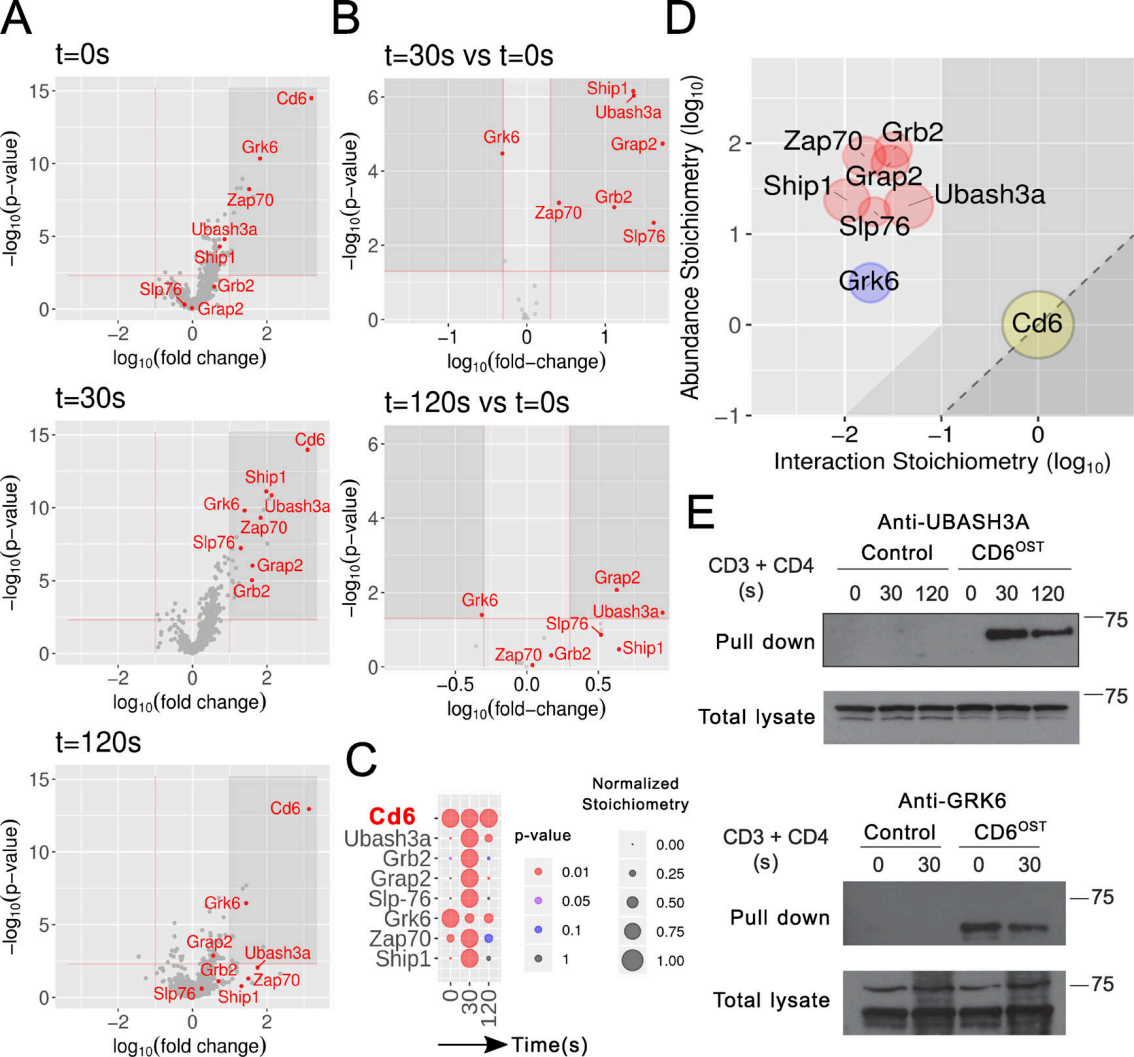
**Composition and dynamics of the CD6 signalosome of long-term expanded CD6^OST^ CD4^+^ T cells.** **(A)** Volcano plot showing proteins significantly enriched after affinity purification in CD4^+^ T cells expressing CD6^OST^ molecules compared with affinity purification in control CD4^+^ T cells expressing similar levels of WT (untagged) CD6 proteins before (t_0s_) and at 30 s (t_30s_) and 120 s (t_120s_) after TCR plus CD4 stimulation. **(B)** Volcano plot showing proteins significantly enriched after affinity purification in CD6^OST^-expressing CD4^+^ T cells 30 and 120 s after TCR engagement compared with affinity purification in unstimulated CD6^OST^-expressing CD4^+^ T cells. For A and B, see description in Fig. 2 B. **(C)** Dot plot showing the interaction stoichiometry over the course of TCR stimulation of CD6 with its seven high-confidence preys, the interaction stoichiometry of which changed following TCR engagement. See description in Fig. 2 C. **(D)** Stoichiometry plot of the CD6 interactome. The CD6 bait is shown as a yellow dot. Red and blue dots correspond to preys that showed increased or decreased binding following TCR stimulation. The purple dot corresponds to a prey whose association was not regulated by TCR stimulation. See description in Fig. 2 D. **(E)** Biochemical validation of the CD6–UBASH3A and CD6–GRK6 interactions predicted on the basis of AP-MS analysis. Long-term–expanded WT (Control) and CD6^OST^-expressing CD4^+^ T cells were left unstimulated (0) or were stimulated for 30 and 120 s with anti-CD3 and anti-CD4 antibodies and subsequently lysed. Equal amounts of cell lysates (Total lysate) were immunoblotted with antibodies specific for UBASH3A and GRK6. Equal amounts of lysates were subjected to AP on Strep-Tactin Sepharose beads (Pull down), followed by elution with D-biotin. Eluates were immunoblotted and probed with antibodies specific for UBASH3A and GRK6. Molecular masses are shown. Data are representative of at least two experiments.

Next, we determined the CD6 interactome of long-term–expanded CD4^+^ T cells edited to express OST-tagged CD6 proteins (Fig. S3, A–C). The appended OST had no detectable effect on CD6 expression (Fig. S3 D) or on the global pattern of TCR-induced tyrosine phosphorylation (Fig. S3 I). Immunoblot analysis of WT and of CD6^OST^-expressing CD4^+^ T cells showed that CD6^OST^ molecules were efficiently purified with Strep-Tactin (Fig. S3 J, lower panel). After stimulation with anti-CD3 and anti-CD4, tyrosine phosphorylation of CD6^OST^ molecules reached a maximum 30 s after stimulation and led to their association with tyrosine phosphorylated species (Fig. S3 J, upper panel). The protein complexes that assembled around CD6^OST^ bait proteins were identified by AP-MS and analyzed as described for the LAT^OST^ bait. 18 interacting proteins showed a >10-fold enrichment, with a P value below 0.005 in at least one of the three conditions of stimulation (Fig. 3 A and Data S2). Among the 18 high-confidence CD6 interactors, 7 showed interaction stoichiometry that increased or decreased at least twofold with a P value below 0.05 following CD3 plus CD4 cross-linkage, compared with the nonstimulated condition (Fig. 3 B). They include the ubiquitin-associated and SH3 domain–containing protein UBASH3A (also known as STS-2 or TULA; Tsygankov, 2020), the G protein–coupled receptor kinase (GRK) 6, SLP-76, GRB2, GRAP2, the ZAP70 PTK, and SHIP1. Six of the seven selected preys showed a transient pattern of binding that peaked 30 s after stimulation, whereas GRK6 showed maximal association to CD6 before TCR stimulation and decreased association thereafter (Fig. 3 C).

The CD6–prey interaction stoichiometries had a narrower distribution than that of the LAT–prey interactions (Fig. 3 D). The previously unreported CD6–UBASH3A and CD6–GRK6 interactions were validated by coimmunoprecipitation of proteins in primary CD6^OST^ CD4^+^ T cells (Fig. 3 E). Consistent with our AP-MS results, UBASH3A interacted with CD6 upon TCR stimulation, whereas GRK6 disassembled in part from CD6 upon TCR engagement. Due to its expression on activated CD4^+^ T cells and micromolar affinity for CD6 (Hassan et al., 2004), ALCAM interacted with CD6 over all the tested conditions and is thus part of the high-confidence constitutive CD6 interactors (Data S2). Likewise, the RHO GTPase–activating protein ARHGAP45 and the α subunit of casein kinase II (CSNK2A1) were also part of the high-confidence constitutive CD6 interactors (Data S2). The presence of CSNK2A1 supports a former study suggesting that serine residues within the CD6 cytoplasmic segment are constitutively phosphorylated by CSNK2 (Bonet et al., 2013). Therefore, the composition and dynamics of the CD6 signalosome revealed a complexity higher than expected.

### The CD5 interactome of short-term–expanded CD4^+^ T cells

**Figure S4.**
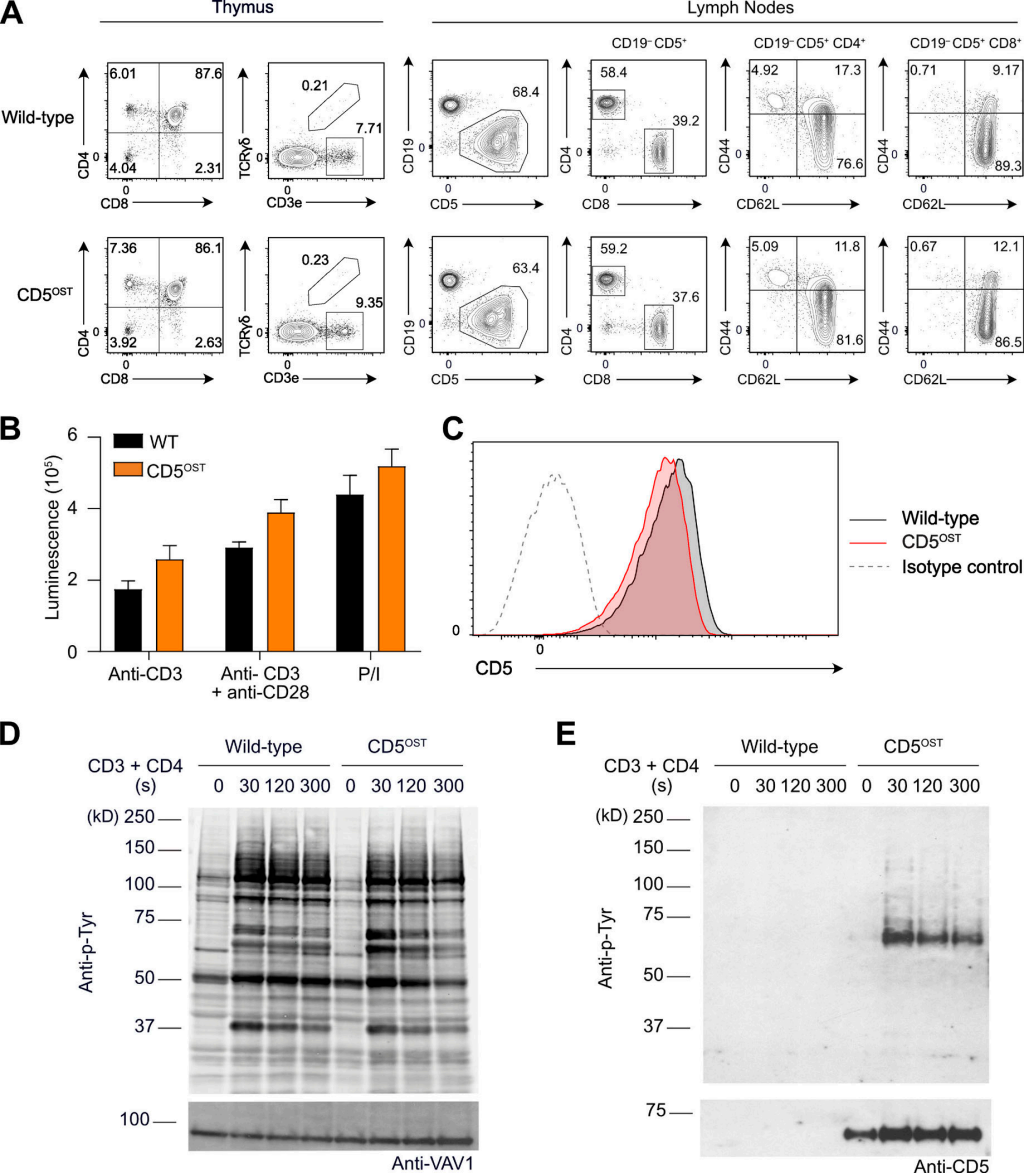
**Normal development and function of T cells of mice expressing endogenous CD5 molecules tagged with an OST tag (CD5^OST^).** **(A)** Flow cytometry analysis of thymus and lymph nodes. WT and CD5^OST^ thymocytes were analyzed for expression of CD4 and CD8 (left) and TCRαβ and TCRγδ (right). Numbers adjacent to outlined areas indicate percentage of CD4^+^CD8^+^ double-positive cells, CD4^+^ single-positive cells, CD8^+^ single-positive cells, and double-negative CD4^−^CD8^−^ cells (left) and of TCRαβ and TCRγδ T cells (right). CD4^+^ (CD19^−^CD5^+^CD4^+^) and CD8^+^ (CD19^−^CD5^+^CD8^+^) T cells from WT and CD5^OST^ lymph nodes were analyzed for CD44 and CD62L expression. Numbers in quadrants indicate percent naive T cells (CD44^low^CD62L^high^) and effector (CD44^high^CD62L^low^) and central (CD44^high^CD62L^high^) memory T cells. Data are representative of at least two experiments with three mice per group. **(B)** Negatively purified CD4^+^ T cells from WT and CD5^OST^ mice were activated in vitro with plate-bound anti-CD3 antibody in the absence or presence of soluble anti-CD28 antibody or with PMA and ionomycin (P/I). After 48 h of culture, ATP content was assessed by luminescence as a measure of the extent of cell proliferation. Data are representative of at least three experiments with two mice per group (mean and SEM are shown). **(C)** Purified CD4^+^ T cells from WT and CD5^OST^ mice were stained with anti-CD5 and analyzed by flow cytometry. Dashed curves: isotype-matched control antibody (negative control). Data are representative of at least three experiments with two mice per group. **(D)** Immunoblot analysis of equal amounts of proteins from total lysates of short-term–expanded T cells from WT and CD5^OST^ mice left unstimulated (0) or stimulated with anti-CD3 plus anti-CD4 for 30, 120, and 300 s and probed with antibody to phosphorylated tyrosine (Anti-p-Tyr) or anti-VAV1 (loading control). **(E)** Immunoblot analysis of equal amounts of proteins from total lysates of cells as in D, subjected to affinity purification on Strep-Tactin Sepharose beads followed by elution of proteins with D-biotin, and probed with antibody to phosphorylated tyrosine (Anti-p-Tyr) or anti-CD5 (affinity purification control). Left margins of D and E: molecular size in kilodaltons. In D and E, data are representative of three independent experiments.

**Figure 4.**
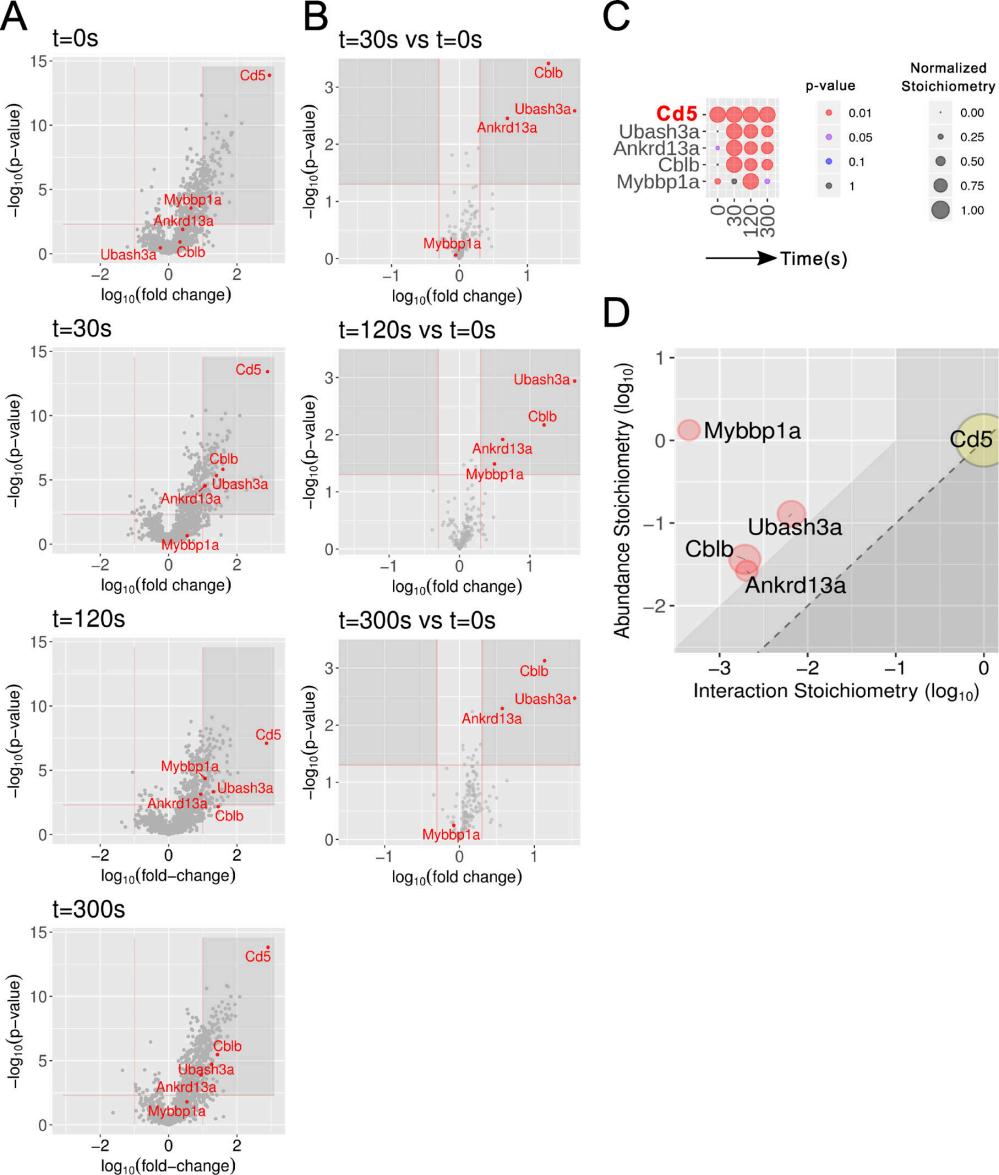
**Composition and dynamics of the CD5 interactome of short-term–expanded CD4^+^ T cells from CD5^OST^ mice.** **(A)** Volcano plot showing proteins significantly enriched after affinity purification in CD4^+^ T cells expressing CD5^OST^ molecules compared with affinity purification in control CD4^+^ T cells expressing similar levels of WT (untagged) CD5 proteins before (t_0s_) and at 30 s (t_30s_), 120 s (t_120s_), and 300 s (t_300s_) after TCR plus CD4 stimulation. **(B)** Volcano plot showing proteins significantly enriched after affinity purification in CD5^OST^-expressing CD4^+^ T cells 30, 120, and 300 s after TCR engagement compared with affinity purification in unstimulated CD5^OST^-expressing CD4^+^ T cells. For A and B, see description in Fig. 2 B. **(C)** The dot plot shows the interaction stoichiometry over the course of TCR stimulation of CD5 with its four high-confidence interactors, the interaction stoichiometry of which changed following TCR engagement. See description in Fig. 2 C. **(D)** Stoichiometry plot of the CD5 interactome. It shows that hundreds of copies of CD5–CBLB, CD5–UBASH3A, and CD5–ANKRD13A complexes formed per T cell as early as 30 s of TCR stimulation, mobilizing close to 10% of the cellular pool of ANKRD13A, CBLB, and UBASH3A. The CD5 bait is shown as a yellow dot. Red dots correspond to preys that show increased binding following TCR stimulation. See description in Fig. 2 D. Mybbp1a; MYB-binding protein 1A.

To identify the composition of the CD5 interactome of primary T cells, we benefited from gene-targeted mice that were available at the onset of our study and that expressed an OST at the C terminus of endogenous CD5 molecules (Fig. S4). Purified primary CD4^+^ T cells from mice expressing endogenous CD5 molecules tagged with an OST tag (CD5^OST^) were expanded for 4 d in vitro to reach the cell numbers required for AP-MS and are subsequently referred to as short-term expanded. The protein complexes assembling around CD5^OST^ bait proteins were identified by AP-MS and analyzed as described for LAT^OST^ and CD6^OST^ baits. 173 interacting proteins showed a >10-fold enrichment, with a P value below 0.005 in at least one of the four conditions of stimulation (Fig. 4 A and Data S2). Among them, four interactors showed interaction stoichiometry that increased at least twofold, with a P value below 0.05 following CD3 plus CD4 cross-linkage, compared with the nonstimulated condition (Fig. 4 B). They comprised the ankyrin repeat domain–containing protein 13A (ANKRD13A), UBASH3A, and CBLB (Fig. 4, C and D). After the fold enrichment value was slightly relaxed, CD5 was also found to interact with CBL (Data S2), a finding consistent with the reciprocal presence of CD5 among high-confidence CBL interactors (Voisinne et al., 2019).

### Function of primary T cells lacking CD5 or CD6 and doubly deficient for CD5 and CD6

**Figure 5.**
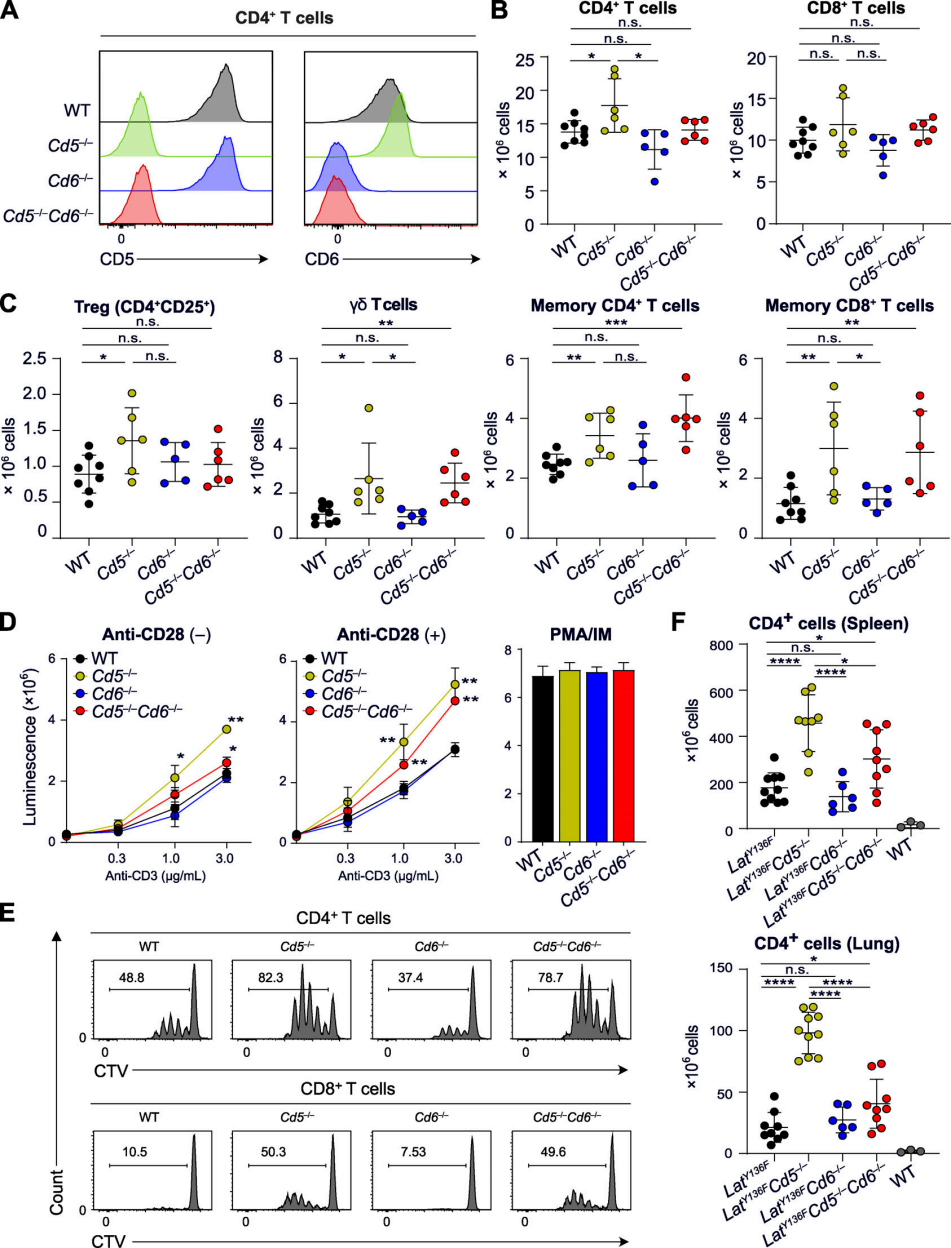
**T cell development and function in CD5-CD6 doubly deficient mice compared with mice lacking either CD5 or CD6.** **(A)** Expression of CD5 and CD6 on CD4^+^ T cells from WT mice and from mice lacking either CD5 (*Cd5^−^*^/−^) or CD6 (*Cd6^−^*^/−^) and both CD5 and CD6 (*Cd5^−^*^/−^*Cd6^−^*^/−^) analyzed by flow cytometry. Note that *Cd6^−^*^/−^ T cells expressed levels of CD5 comparable to that of WT mice, whereas increased levels of CD6 were found on *Cd5^−^*^/−^ T cells as reported (Orta-Mascaró et al., 2016). **(B)** Numbers of CD4^+^ and CD8^+^ T cells found in the spleen of the specified mice. **(C)** Numbers of T reg cells, γδ T cells, and effector memory CD4^+^ and CD8^+^ T cells found in the spleen of the specified mice. **(D)** T cells were purified by immunomagnetic negative selection from lymph nodes of *Cd5^−^*^/−^, *Cd6^−^*^/−^, *Cd5^−^*^/−^*Cd6^−^*^/−^, and WT mice activated in vitro with the specified concentrations of plate-bound anti-CD3 in the absence (−) or presence (+) of soluble anti-CD28 (1 µg/ml). After 48 h of culture, ATP content was assessed using luminescence as a measure of the extent of cell proliferation. The histogram on the right shows the extent of cell proliferation in response to PMA plus ionomycin (PMA/IM). **(E)** Profiles of CTV-labeled CD4^+^ (upper panel) and CD8^+^ (lower panel) T cells isolated from the specified mice and stimulated with anti-CD3 plus anti-CD28 antibodies for 72 h. The percentages of proliferating CTV^low^ T cells are indicated. **(F)** Numbers of CD4^+^ T cells found in the spleen and lungs of WT, *Lat*^Y136F^, *Lat*^Y136F^ *Cd5^−^*^/−^, *Lat*^Y136F^ *Cd6^−^*^/−^, and *Lat*^Y136F^ *Cd5^−^*^/−^*Cd6^−^*^/−^ mice. Data in A–E are representative of at least three independent experiments, whereas data in F correspond to the pool of three independent experiments. In B and C, each dot corresponds to a mouse, and the mean (horizontal bar) is indicated. n.s., nonsignificant; *, P ≤ 0.05; **, P ≤ 0.01; ***, P ≤ 0.001; ****, P ≤ 0.0001. Error bars correspond to the mean and SD.

The markedly different composition of the CD5 and CD6 signalosomes led us to analyze in parallel and on a homogenous genetic background the functional consequences resulting from their respective ablation. Accordingly, *Cd5*^−/−^ mice (Tarakhovsky et al., 1995) were backcrossed on a C57BL/6 background, and we developed C57BL/6 mice lacking CD6 (*Cd6*^−/−^) and both CD5 and CD6 (*Cd5*^−/−^*Cd6*^−/−^; Fig. 5 A). Deletion of CD5 or CD6 had few effects on the selection of thymocytes expressing the polyclonal TCR repertoire (Azzam et al., 2001; Orta-Mascaró et al., 2016). Along that line, the thymus of *Cd5*^−/−^, *Cd6*^−/−^, and *Cd5*^−/−^*Cd6*^−/−^ mice showed a normal sequence of T cell development, and slight differences were only noted in the frequency of CD69^+^CD4^+^CD8^+^ cells in the *Cd5*^−/−^ and *Cd6*^−/−^ thymus compared with the WT thymus (not depicted). The cellularity of the secondary lymphoid organs of *Cd5*^−/−^, *Cd6*^−/−^, and *Cd5*^−/−^*Cd6*^−/−^ mice was comparable to that of WT counterparts, and their T cells expressed normal levels of CD3, CD4, and CD8 (Fig. 5 B and not depicted). Slightly increased numbers of CD4^+^ T cells, regulatory T cells (T reg cells), γδ T cells, and memory T cells were found in the secondary lymphoid organs of *Cd5*^−/−^ mice (Fig. 5 C).

**Figure S5.**
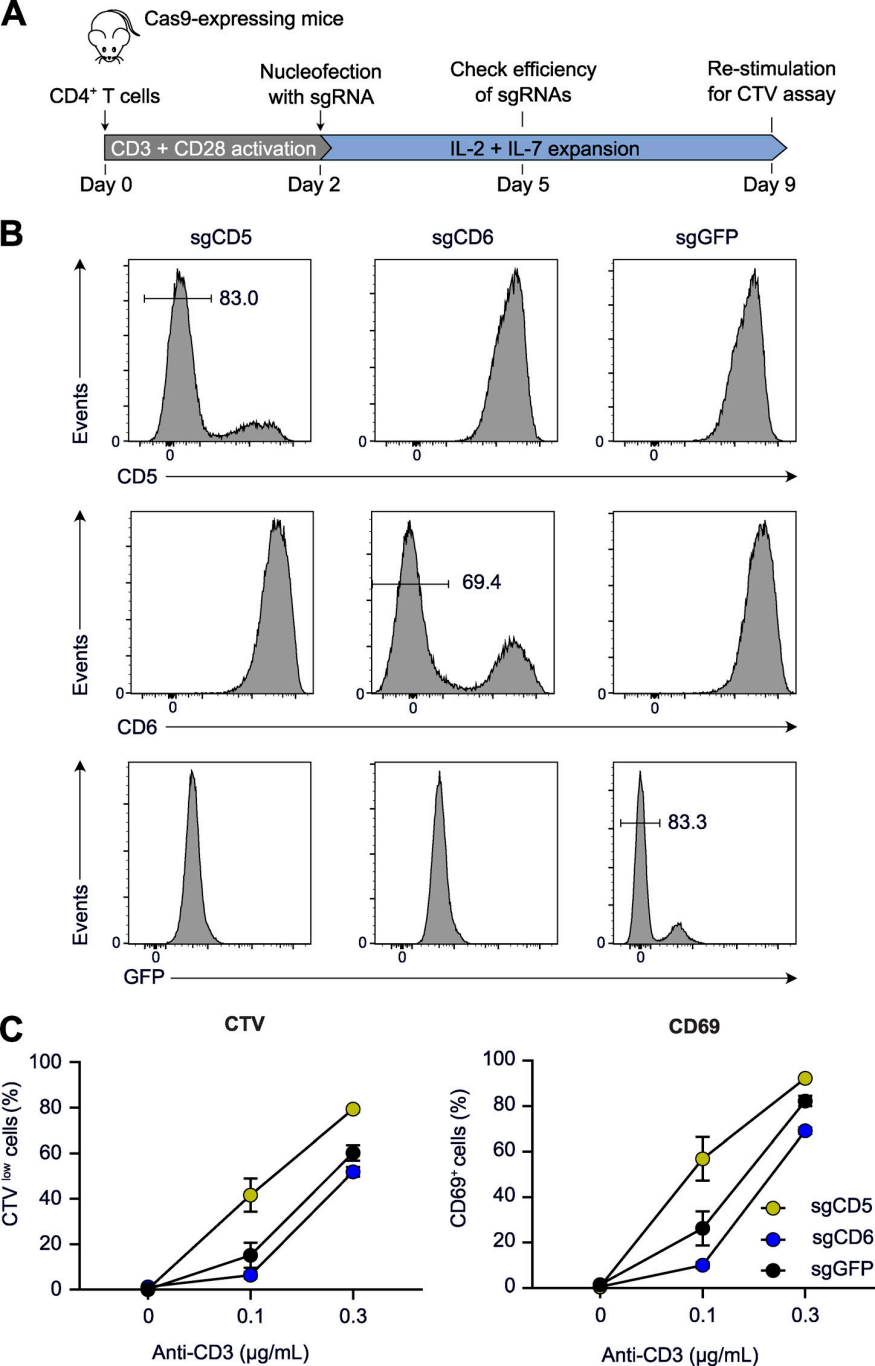
**Augmented TCR-mediated activation in primary WT CD4^+^ T cells rendered CD5 deficient.** **(A)** Workflow used for CD5, CD6, or EGFP gene deletion in CD4^+^ T cells from Cas9-expressing mice. T cells from Cas9-expressing mice (Platt et al., 2014) also express EGFP and were used as control after EGFP gene inactivation. **(B)** Expression of CD5, CD6, and EGFP on CD4^+^ T cells from Cas9-expressing mice nucleofected with sgRNA targeting CD5, CD6, or EGFP (see Table S1). Cells were analyzed by flow cytometry 3 d after nucleofection, and histograms show the levels of CD5, CD6, and EGFP on CD4^+^ T cells nucleofected with the specified sgRNA. Percentages of EGFP^−^, CD5^−^, and CD6^−^ cells are shown. **(C)** Percentages of CTV^low^ (left) and CD69^+^ (right) cells in CD4^+^ T cells nucleofected with sgCD5^−^, sgCD6^−^, or sgGFP 7 d before and stimulated for 48 h with the specified concentrations of plate-bound anti-CD3 in the presence of soluble anti-CD28 antibodies (1 µg/ml). Data are representative of at least two independent experiments. Error bars correspond to the mean and SD. Sg, single guide.

T cells from *Cd5*^−/−^, *Cd6*^−/−^, *Cd5*^−/−^*Cd6*^−/−^, and WT mice were activated in vitro with plate-bound anti-CD3 in the presence or absence of anti-CD28. The lack of CD6 had no measurable effect on either TCR- or TCR plus CD28–induced proliferation (Fig. 5 D). In contrast, the lack of CD5 enhanced proliferation at all the tested CD3 concentrations and regardless of CD28 engagement (Fig. 5 D). Comparison of *Cd5*^−/−^ and *Cd5*^−/−^*Cd6*^−/−^ mice showed that the enhanced proliferation resulting from the lack of CD5 was diminished by the absence of CD6. These conclusions were confirmed using the division tracking dye CellTrace Violet (CTV; Fig. 5 E). Deleting CD5 in primary T cells isolated from WT mice enhanced their TCR-induced proliferation compared with that of control cells, whereas CD6 deletion had no measurable effect (Fig. S5). Therefore, the enhanced TCR responses of T cells isolated from *Cd5*^−/−^ mice did not result from adaptive mechanisms set in motion during T cell development to compensate for the lack of CD5.

Mutant mice in which tyrosine at position 136 of LAT is replaced with phenylalanine (*Lat*^Y136F^ mice) develop a lymphoproliferative disorder involving CD4^+^ T cell effectors that trigger systemic autoimmunity (Aguado et al., 2002). The lack of CD6 was found without measurable effect on the unfolding of the *Lat*^Y136F^ pathology, whereas the lack of CD5 exacerbated it, resulting in 2.4-fold increased numbers of pathogenic CD4^+^ T cells in the spleen (Fig. 5 F). In the absence of CD5 and CD6, the numbers of pathogenic *Lat*^Y136F^ CD4^+^ T cells ranked between those observed in *Cd5*^−/−^ and WT mice. Dense perivascular infiltrates consisting of pathogenic CD4^+^ T cells are present in the lungs of *Lat*^Y136F^ mice (Genton et al., 2006). Although *Cd6*^−/−^ T cells showed impaired infiltration through brain microvascular endothelial cell monolayers (Li et al., 2017), the lack of CD6 was without effect on the magnitude of the lung infiltrate, whereas that of CD5 resulted in fourfold-increased numbers of infiltrating cells (Fig. 5 F). In the absence of CD5 and CD6, infiltrating CD4^+^ T cell numbers ranked between those observed in *Cd5*^−/−^ and WT mice. Altogether, these results showed that the absence of CD5 rendered T cells more reactive to TCR and CD28 triggering and increased the numbers of pathogenic *Lat*^Y136F^ T cells in both lymphoid and nonlymphoid organs. Therefore, CD5 acts as a negative regulator of T cell activation regardless of the presence of CD6. In contrast, it is only in the context of a CD5 deficiency that we succeeded in documenting a net CD6 costimulatory effect (Fig. 5, D–F).

### LAT differs from CD5 and CD6 in that it triggers most TCR/CD28–induced transcription-dependent events

**Figure 6.**
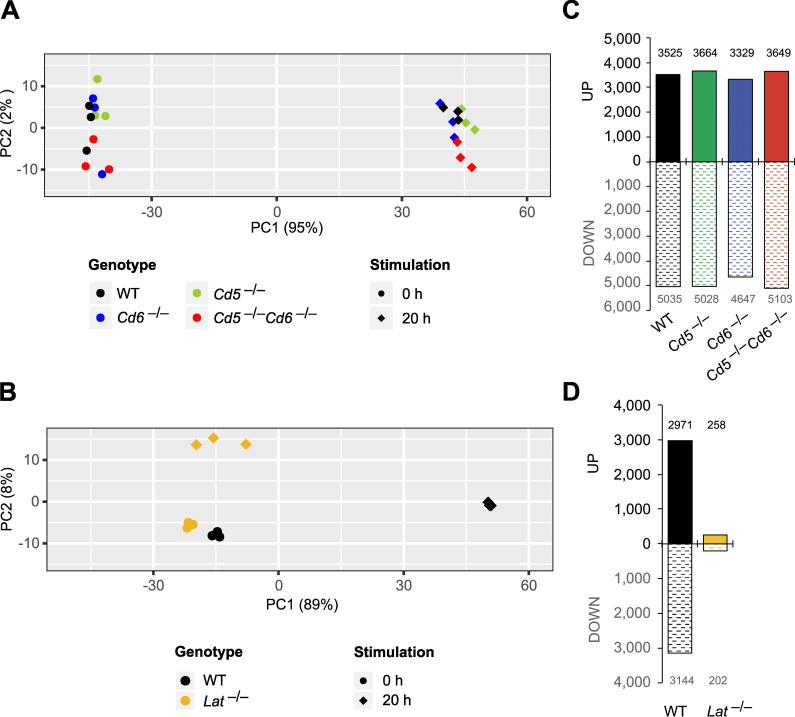
**Transcriptional changes occurring in primary CD4^+^ T cells after engaging the TCR–CD28 pathways in the absence of LAT, of CD5, of CD6, or of both CD5 and CD6.** **(A)** PCA of gene expression in WT, *Cd5^−^*^/−^, *Cd6^−^*^/−^, and *Cd5^−^*^/−^*Cd6^−^*^/−^ primary CD4^+^ T cells that were left unstimulated (0 h) or stimulated for 20 h with anti-CD3 plus anti-CD28. **(B)** PCA of gene expression in WT and *Lat^−^*^/−^ primary CD4^+^ T cells that were left unstimulated (0 h) or stimulated for 20 h with anti-CD3 plus anti-CD28 (20 h). **(C)** Quantification of the genes significantly induced (UP) or repressed (DOWN) in WT, *Cd5^−^*^/−^, *Cd6^−^*^/−^, and *Cd5^−^*^/−^*Cd6^−^*^/−^ CD4^+^ T cells after stimulation for 20 h with anti-CD3 plus anti-CD28. **(D)** Quantification of the genes significantly induced (UP) or repressed (DOWN) in WT and *Lat^−^*^/−^ CD4^+^ T cells after stimulation for 20 h with anti-CD3 plus anti-CD28. See Data S3 for details on the induced or repressed genes quantified in C and D. In A–D, three independent samples were prepared for each condition. In A and B, numbers shown in parentheses on the PCA1 and PCA2 axes indicate the percentage of overall variability in the dataset along each PC axis. *Lat^−^*^/−^ CD4^+^ T cells were obtained using *Lat*^fl-dtr^ maT-Cre mice (Roncagalli et al., 2014).

To compare the respective contribution of LAT, CD5, and CD6 with the transcriptional changes elicited by TCR-CD28 stimulation, WT, *Lat*^−/−^, *Cd5*^−/−^, *Cd6*^−/−^, and *Cd5*^−/−^*Cd6*^−/−^ naive CD4^+^ T cells were sorted and subjected to RNA-sequencing analysis before or after stimulation for 20 h with anti-CD3 and anti-CD28. Using principal component analysis (PCA), TCR-CD28 activated *Cd5*^−/−^, *Cd6*^−/−^, and *Cd5*^−/−^*Cd6*^−/−^ CD4^+^ T cells clustered with TCR-CD28–stimulated WT cells (Fig. 6 A), suggesting that the bulk of TCR-CD28–induced transcriptional changes occurred unabated in the absence of CD5, CD6, or both CD5 and CD6. In contrast, anti-CD3 plus anti-CD28 activated *Lat*^−/−^ CD4^+^ T cells clustered close to unstimulated WT and *Lat*^−/−^ CD4^+^ T cells (Fig. 6 B), indicating that the absence of LAT blunted most TCR-CD28–induced transcriptional events, as previously hinted at using microarray analysis (Roncagalli et al., 2014). Consistent with PCA, the number of genes differentially expressed (fold change >2 and adjusted P value <0.05) between unstimulated and TCR-CD28–stimulated *Cd5*^−/−^, *Cd6*^−/−^, and *Cd5*^−/−^*Cd6*^−/−^ CD4^+^ T cells was comparable to that of WT CD4^+^ T cells (Fig. 6 C and Data S3). In contrast, the number of genes differentially expressed between unstimulated and TCR-CD28–stimulated cells was dramatically decreased in *Lat*^−/−^ CD4^+^ T cells compared with WT CD4^+^ T cells (Fig. 6 D and Data S3). Therefore, our transcriptomics results are consistent with the functional outcomes resulting from the ablation of LAT, CD5, and CD6, in that LAT-deficient T cells failed to proliferate and produce cytokines in response to TCR-CD28 stimulation (Mingueneau et al., 2009) and thus markedly differ from T cells deprived of CD5 and CD6, which responded comparably (CD6^−/−^) or slightly better (CD5^−/−^) than WT T cells did (Fig. 5).

### Inefficient assembly of high-order LAT signalosomes

**Figure 7.**
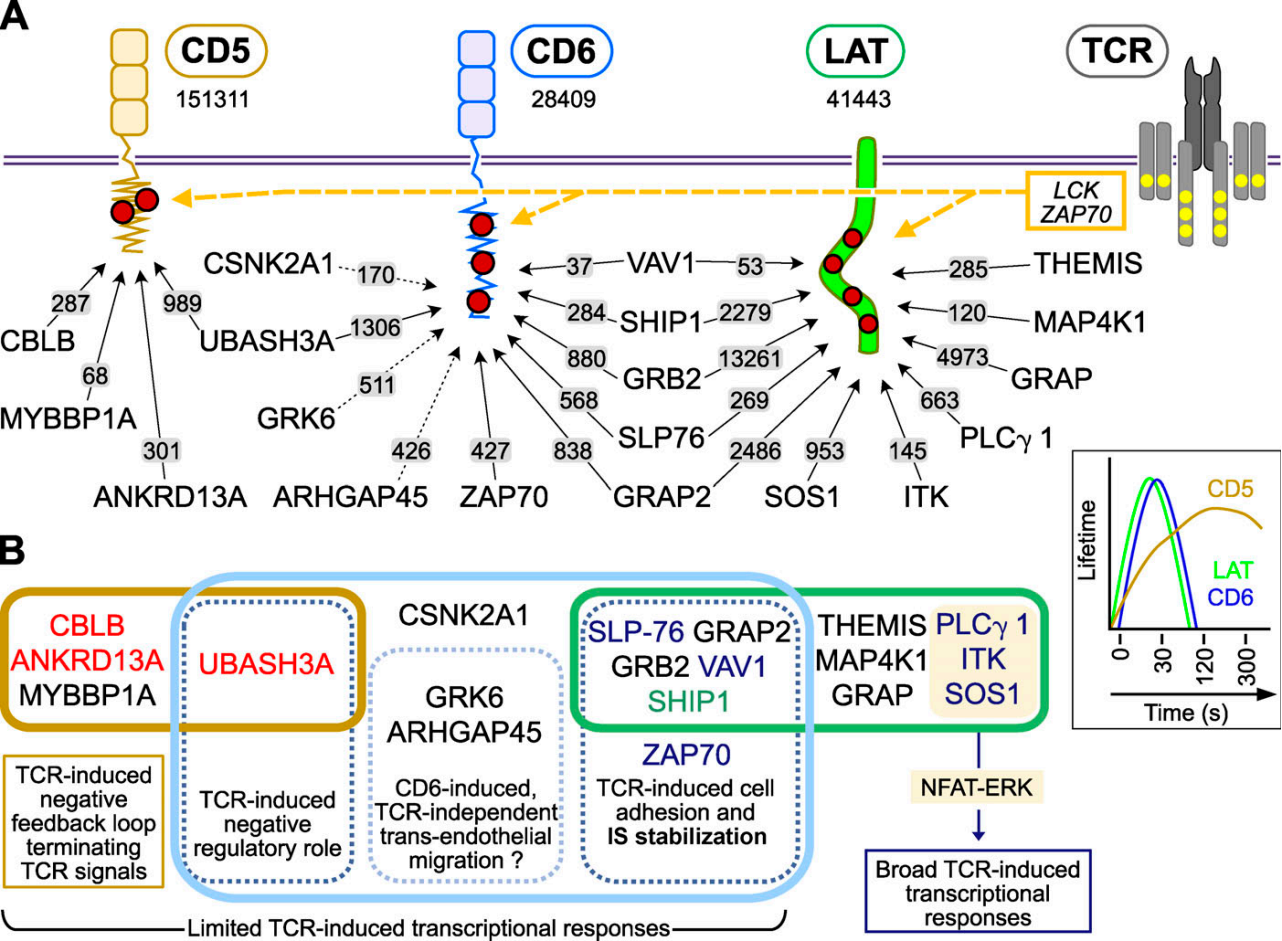
**A quantitative model of early TCR signal diversification integrating interactomics and transcriptomics.** **(A)** Tyrosine residues (red dots) present in the intracytoplasmic segments of CD5, CD6, and LAT are phosphorylated by the LCK or ZAP70 PTK that associate with active TCR (dashed yellow arrows). Following 30 s of TCR engagement, distinct signalosomes nucleated around the tyrosine phosphorylated CD5 and CD6 transmembrane receptors and the LAT transmembrane adaptor. The number of copies per T cell of CD5, CD6, and LAT is indicated. For instance, 41,443 copies of LAT are expressed per T cell. The maximum copies per T cell of high-confidence bait–prey complexes reached over the course of TCR stimulation is also shown and specified over the arrows connecting the baits and the preys. For instance, the maximum number of CD5–UBASH3A complexes reached per T cell over the course of TCR stimulation is ∼989 (see Fig. 2 D legend and Data S2). Solid arrows correspond to TCR-induced interactions, whereas dotted arrows correspond to constitutive interactions. Consistent with former studies (Roncagalli et al., 2014; Voisinne et al., 2019), after cutoff values were slightly relaxed, VAV1 was found to interact significantly and in a TCR-inducible manner with both CD6 (5.0-fold enrichment; P value: 3.2 × 10^−5^) and LAT (8.5-fold enrichment; P value: 4.8 × 10^−6^), reaching in both cases maximum binding 30 s after TCR engagement (Data S2). CD5 showed a transient binding with MYB-binding protein 1A (MYBBP1A), a transcriptional regulator that shuttles between the cytoplasm and the nucleus, and the role within the CD5 interactome remains to be determined. Inset: Lifetime of the TCR-induced interactions involving CD5, CD6, and LAT with most of their interactors. **(B)** The Venn diagram illustrates the commonalities and differences between the CD5, CD6, and LAT signalosomes as well as the functional outcomes expected to result from their engagement at the IS.

The global picture of the LAT signalosome obtained using LAT as a bait (Fig. 7 A) is fully consistent with the “reciprocal” picture inferred from the use of 15 baits corresponding to canonical proteins of the TCR-signaling pathway (Voisinne et al., 2019), and it supports the LAT model obtained by addressing one interactor at a time (Balagopalan et al., 2010). However, the possibility of enumerating the number of each LAT–prey interaction that forms per T cell provided an unprecedented quantitative picture of the LAT signalosome (Fig. 7 A and Data S2). Among the GRB2 protein family, GRAP formed 4,973 interactions with LAT 30 s after TCR stimulation, compared with 13,261 and 2,486 for GRB2 and GRAP2, respectively. Although GRAP has received less attention than GRB2 and GRAP2 in the context of LAT (Trüb et al., 1997), its important quantitative contribution to the LAT signalosome emphasizes the pressing need to determine the constellation of molecules it plugs into the LAT signalosome via its SH3 domains. LAT and SLP-76 interact via GRAP2 intermediate, whereas LAT–THEMIS, LAT–SOS1, and LAT–SHIP1 interactions occur through GRB2 intermediate. Prior to TCR engagement, GRAP2–SLP76, GRB2–SHIP1, GRB2–SOS1, and GRB2–THEMIS form stable binary complexes, leaving a large fraction of GRAP2 and GRB2 molecules in a “free” form or in complex with unknown partners (Voisinne et al., 2019). When the LAT signalosome reached its maximal interaction stoichiometry, the sum of the LAT–SLP-76, LAT–SHIP1, LAT–SOS1, and LAT–THEMIS interactions was fourfold smaller than that of LAT–GRAP2 and LAT–GRB2 interactions (Fig. 7 A). The possibility that GRAP2 and GRB2 combine with unknown partners might explain the fourfold excess of LAT–GRAP2 and LAT–GRB2 interactions over LAT–THEMIS, LAT–SOS1, and LAT–SHIP1 interactions. However, owing to the advanced inventory of GRAP2 and GRB2 interactors, such excess is more likely due to the competition that exists between the GRAP2–SLP-76, GRB2–SHIP1, GRB2–SOS1, and GRB2–THEMIS complexes and the larger pool of free GRAP2 and GRB2 adaptors for binding to phosphorylated LAT molecules. Therefore, the formation of a larger number of abortive or partially functional LAT signalosomes involving uncomplexed members of the GRB2 protein family likely accompanied that of higher-order and fully functional LAT signalosomes. Interestingly, the maximum numbers of LAT–SLP-76 and LAT–SHIP1 interactions that were reached 30 s after TCR engagement corresponded to 269 and 2,279 copies per T cell, respectively (Fig. 7 A), suggesting that a majority of SHIP-containing LAT signalosomes lack SLP-76 and pointing to the existence of several LAT signalosome isoforms that can further assemble into condensates (Houtman et al., 2006; Huang et al., 2016; Malissen et al., 2014).

### Quantitative model of the CD5 signalosome

In the absence of GRB2 in the CD5 interactome (Data S2), CBLB likely binds to tyrosine-phosphorylated CD5 molecules via its tyrosine kinase–binding domain. Upon colocalization with the TCR at IS (Brossard et al., 2003), CD5-bound CBLB molecules are tyrosine phosphorylated by TCR-operated PTK. In turn, their RING-type zinc finger domain becomes available for binding to E2 ubiquitin–conjugating enzymes, leading to K63 ubiquitylation of neighboring substrates that may include CD5 and CBLB themselves (Demydenko, 2010; Voisinne et al., 2016). The K63 ubiquitylated complex assembling around the CD5-CBLB seed allows the recruitment of ANKRD13A via its K63-specific ubiquitin-interacting motifs (Fig. 7 A). ANKRD13A regulates endocytosis of K63-ubiquitylated forms of the epidermal growth factor receptor (Tanno et al., 2012) and of the B cell antigen receptor (Satpathy et al., 2015) through its interaction with the endocytic machinery.

Along the same line, ANKRD13A might contribute to the endocytosis of CD5-CBLB–containing vesicles. In addition to its E3 ubiquitin–protein ligase activity, CBLB possesses numerous interactive elements, including a proline-rich region that constitutively binds the SH3 domain of UBASH3A (Feshchenko et al., 2004; Ge et al., 2019; Voisinne et al., 2019) and likely accounts in part for the recruitment of UBASH3A to the TCR-induced CD5 signalosome. T cells deficient for both UBASH3A and its UBASH3B paralog (also known as STS-1 or TULA-2) are hyper-responsive to TCR engagement, a finding consistent with their increased ZAP70 activity (Carpino et al., 2004). Although UBASH3A possesses reduced phosphatase activity compared with that of UBASH3B, its negative regulatory function may involve its SH3 and UBA domains (San Luis et al., 2011). Further support for an inhibitory function of UBASH3A in primary T cell activation was recently provided by a study demonstrating that upon association with the CIN85 adaptor, UBASH3A was recruited to TCR microclusters and inhibited their signals (Kong et al., 2019). Therefore, our identification of UBASH3A within the TCR-induced CD5 signalosome of primary T cells in addition to that of CBLB further explains its negative regulatory function.

### Quantitative model of the CD6 signalosome

Two types of CD6–prey interactions can be distinguished among the CD6 signalosome. Some existed before TCR engagement and persisted (ARHGAP45) or decreased (GRK6) over 120 s of stimulation, whereas others occurred in a TCR-inducible manner. Among these last interactions, a first set involved interactors (ZAP70, SLP-76, and VAV1) endowed with positive regulatory functions. ZAP70 is mandatory for the LAT-independent, TCR-inducible assembly of the CD6 signalosome (Roncagalli et al., 2014) and, together with VAV1, likely contributes to IS stabilization via integrin activation (Jenkins et al., 2014; Meddens et al., 2018; Roncagalli et al., 2014; Zimmerman et al., 2006). The adaptors GRB2, GRAP2, and SLP-76 also associated with CD6 in a TCR-inducible manner, likely contributing to recruitment of VAV1 and SHIP1. A second set of TCR-inducible CD6 interactors (UBASH3A and SHIP1) is endowed with negative regulatory functions. By catalyzing the hydrolysis of the phosphatidylinositol 3-kinase product PI(3,4,5)P_3_ into PI(3,4)P_2_, SHIP1 negatively regulates positive effectors with pleckstrin homology domain specific for PI(3,4,5)P_3_. Therefore, negative regulatory functions can also be conferred to CD6 via its interaction via SHIP1. However, this view needs to be mitigated since SHIP1 can also set in action positive effectors containing a pleckstrin homology domain specific for PI(3,4)P_2_, including protein kinase B (AKT) isoforms (Liu et al., 2018). Importantly, among the documented CD6–prey interactions, the CD6–UBASH3A interaction ranked first in terms of numbers of copies per T cell (Fig. 7 A). Therefore, in contrast to the CD5 signalosome, the CD6 signalosome comprised TCR-inducible interactors endowed with both positive and negative functions, the dominance of which likely depends on the immune response being measured (Gonçalves et al., 2018). However, our AP-MS analysis does not permit exclusion of the existence of distinct CD6 signalosome isoforms with either a negative or a positive regulatory role.

## Discussion

We developed a fast-track interactomics approach permitting assessment of the composition and dynamics of signalosomes assembling in primary T cells in response to TCR stimulation. Here, we focused on the LAT, CD5, and CD6 signalosomes since they coincidently assemble with fast kinetics following TCR engagement (Voisinne et al., 2019). We demonstrated that SLP-76 is part of the CD6 and LAT signalosomes, a finding implying that our former use of SLP-76 as a bait led to the copurification of both CD6 and LAT signalosomes (Voisinne et al., 2019). Based on the present study, SLP-76 interactors such as UBASH3A and PLC-γ1 can be assigned to the CD6 and LAT signalosomes, respectively, whereas VAV1 belongs to both of them. Therefore, disentangling the intricacy of the signal-transduction networks involved in T cell activation and of the underlying signalosomes requires conducting an AP-MS analysis at multiple entry points corresponding to both T cell surface receptors and their cytosolic effectors. Note that SLP-76 differently exploits its scaffolding potential to bind to LAT and CD6. The SLP-76–LAT interaction occurs via GRAP2 alone, whereas the SLP-76–CD6 interaction involves the SH2 domain of both GRAP2 and SLP-76. Such distinct modes of binding have important functional implications in that it is solely when SLP-76 is complexed to LAT that its SH2 domain is free to interact with MAP4K1, leading to the dismantling of the LAT signalosome via 14–3-3 proteins.

The possibility of combining time-resolved AP-MS analysis of the LAT and SLP-76 signalosomes also provided a fine-grained view of their dismantling. 120 s after TCR triggering, SLP-76 was totally disconnected from LAT and only interacting with 14–3-3 proteins, which promoted SLP-76 detachment from LAT (Voisinne et al., 2019). However, part of the LAT signalosome might have remained intact and functional. Using LAT as a bait, we confirmed here that 120 s after TCR triggering, LAT–SLP-76 interactions were fully disrupted and further showed that LAT itself was stripped of most of its other interactors. Consistent with previous studies (Hashimoto-Tane and Saito, 2016), the LAT signalosomes that are present 30 s after TCR engagement are thus almost gone 90 s later. The present study also allowed analysis of the extent of proteome remodeling that occurs during long-term CD4^+^ T cell expansion. Comparing the fraction of cell mass occupied by the proteins quantified in short-term– and long-term–expanded CD4^+^ T cells showed that the fraction of cell mass occupied by 79% of the quantified proteins differed by less than fourfold between short-term– and long-term–expanded CD4^+^ T cells (Fig. S2).

The numbers of CD6–SLP-76 and LAT–SLP-76 interactions can be used as a proxy of active CD6 and LAT signalosomes, respectively. These numbers peaked 30 s after TCR engagement and differed by approximately twofold. Despite this rather similar quantitative contribution to TCR signal propagation, our transcriptomics analysis demonstrated that LAT markedly differed from CD6 in that it mediated most of the TCR-CD28–induced transcriptional responses, a finding that correlates with the presence of SOS1 and PLC-γ1 in and only in the LAT signalosome and, in turn, with their ability to plug into the ERK and NFAT pathways, respectively (Fig. 7 B). Considering that LAT signalosomes are mostly dismantled 120 s after TCR engagement, it is paradoxical that the CD5–CBLB–UBASH3A–ANKRD13A inhibitory complex persisted up to 300 s after TCR engagement. Provided that the antigen-recognition and triggering module made of TCR-CD3, LCK, and ZAP70 delivers longer lasting signals than LAT signalosomes (Yudushkin and Vale, 2010), this module might constitute the primary target of the CD5–CBLB–UBASH3A complex (Hu et al., 2016; Huang et al., 2010; Ivanova and Carpino, 2016; Yang et al., 2015), thereby preventing further formation of LAT signalosomes. Although the molecular targets of the TCR-inducible CD5–CBLB–UBASH3A multimolecular complex remain to be determined, the observation that CD4^+^ T cells deficient in CD5 (Tarakhovsky et al., 1995; this study), CBLB (Chiang et al., 2007; Paolino et al., 2011), or UBASH3A (Kong et al., 2019) showed enhanced TCR responses strongly supports the negative regulatory function of the CD5–CBLB–UBASH3A signalosome during TCR-mediated T cell activation.

T cells deprived of CD6 have reduced ability to migrate through brain microvascular endothelial cell monolayers (Li et al., 2017), and upon binding to CD6, CD318 constitutes a chemoattractant for T cells (Enyindah-Asonye et al., 2017). These observations likely account for attenuated experimental autoimmune encephalomyelitis severity in the CD6-deficient mouse and the beneficial use of anti-CD6 antibodies in multiple sclerosis treatment (Consuegra-Fernández et al., 2018). T cell transendothelial migration (TEM) is guided by G protein–coupled receptors (GPCRs) specific for chemokines, and liganded GPCRs undergo phosphorylation by GRKs, the functional effect of which appears to be cell-type specific (Steury et al., 2017). Among the novel proteins identified in the CD6 interactome, the presence of GRK6 and ARHGAP45 supports the involvement of CD6 in TEM. GRK6 is expressed at high levels in immune cells, where it regulates chemotaxis of T and B cells in TEM assays (Fong et al., 2002), and ARHGAP45 is a RHO GTPase–activating protein also involved in TEM (de Kreuk et al., 2013; Voisinne et al., 2019). Therefore, the presence of GRK6 and ARHGAP45 in the CD6 interactome irrespective of TCR engagement might explain the role of CD6 in immunopathologies involving TEM and some of the benefits resulting from clinical trials targeting CD6 (Consuegra-Fernández et al., 2018).

In conclusion, using a fast-track systems-level approach we retrieved quantitative information on the multiple TCR-dependent signaling processes that unfold simultaneously at the inner face of the plasma membrane of primary T cells. We provided a quantitative model describing the multitude of signals delivered via LAT molecules expressed at physiological levels in primary T cells following TCR engagement and explaining its unique role in ensuing transcriptional activation. T cell–surface receptors are generally categorized as costimulators or coinhibitors. Along that line, the CD5 signalosome of primary T cells comprised molecules involved in signal termination and routing through the endocytic pathway and can thus be unambiguously defined as a T cell coinhibitor. In contrast, the CD6 signalosome comprised effectors involved in TEM as well as positive and negative regulators of T cell activation. Finally, it remains to be determined whether the intriguing presence of UBASH3A within the CD6 signalosome provides a molecular basis for the observation that single-nucleotide polymorphisms within both CD6 and UBASH3A genes are associated with autoimmune diseases (De Jager et al., 2009; Ge et al., 2019).

## Materials and methods

### Mice

Mice were on a C57BL/6 background, were 8–10 wk old, and were maintained in specific pathogen–free conditions. Mice constitutively expressing Cas9 (Platt et al., 2014) were purchased from the Jackson Laboratory (Gt(ROSA)26Sortm1.1(CAG-cas9*,-EGFP)^Fezh/J^; JAX stock no. 024858). *Lat*^Y136F^ and *Lat*^fl-dtr^ mice (B6;129-*Lat*^tm6Mal^) have been described (Aguado et al., 2002; Mingueneau et al., 2009). CD5-deficient mice (Tarakhovsky et al., 1995) backcrossed on a C57BL/6 background were provided by F. Lozano (Universitat de Barcelona, Barcelona, Spain). Generation of CD6-deficient mice (C57BL/6JRj-*Cd6*^em1Ciphe^), CD5-CD6 doubly deficient mice (C57BL/6JRj-Del(Chr19 *Cd5-Cd6*)^em1Ciphe^), and CD5^OST^ (C57BL/6NRj-*Cd5*^tm1Ciphe^) mice is described below.

### Animal experimental guidelines

Mice were handled in accordance with national and European laws for laboratory animal welfare and experimentation (European Economic Community Council Directive 2010/63/EU, September 2010) and protocols approved by the Marseille Ethical Committee for Animal Experimentation.

### Generation of CD6-deficient mice and of CD5-CD6 doubly deficient mice

Mice doubly deficient in CD5 and CD6 were constructed by using two pairs of sgRNA (Thermo Fisher Scientific) targeting the 5′ end of exon 1 of the *Cd5* gene (5′-CCA​TGG​ACT​CCC​ACG​AAG​TGC​TG-3′) and the beginning of the intron flanking the 3′ end of exon 1 of the *Cd6* gene (5′-CCA​TCG​GTG​GCA​CAA​CCG​TCT​CC-3′). Both were designed using the Crispor algorithm (Haeussler et al., 2016). One-cell C57BL/6JRj embryos at day 0.5 (Janvier Labs) were defrosted and subjected to pronuclear microinjection with the two sgRNA pairs (each used at 5 ng/ml) and 10 ng/ml Cas9 RNA (Thermo Fisher Scientific) in DNase- and RNase-free micro-injection buffer (10 mM Tris and 1 mM EDTA, pH 8; Qiagen). Injected embryos were transferred into the oviducts of day 0.5 pseudopregnant B6/CBA recipient female mice. Genomic DNA from F0 mice was prepared from tail tissue using a solution composed of 100 mM Tris-HCl, pH 7.5, 5 mM EDTA, 200 mM NaCl, 0.2% SDS, 0.5 mg/ml proteinase K, and 50 µg/ml RNaseA and analyzed by PCR using a QIAxcel Advanced System. F0 mice with the expected mutation were further crossed, and the exact boundary of the intended deletion was determined via PCR amplification and sequencing using 100 ng of genomic DNA. A deletion extending from nucleotide 10738791 to nucleotide 10829931 of chromosome 19 (https://www.ensembl.org/Mus_musculus/) was selected and used to establish mice deprived of both CD5 and CD6. These *Cd5*^−/−^*Cd6*^−/−^ mice (also known as C57BL/6JRj-Del(Chr19 Cd5-Cd6)^em1Ciphe^) were born at expected Mendelian frequencies and lacked detectable CD5 and CD6 expression (Fig. 5 A). Mice deficient for CD6 were generated as described above for *Cd5*^−/−^*Cd6*^−/−^ mice. Two sgRNAs were designed to target the 5′ untranslated region (UTR; 5′-CCA​TCG​GTG​GCA​CAA​CCG​TCT​CC-3′) and the start codon (5′-CCA​GAC​ATG​TGG​CTC​TTC​CTT​GG-3′) of the *Cd6* gene. A resulting 170-bp-long deletion extending from nucleotide 10829689 to nucleotide 10829860 of chromosome 19 (https://www.ensembl.org/Mus_musculus/) and encompassing 3 bp of the 5′ UTR and the first 118 bp of exon 1, including the start codon, was selected to establish CD6-deficient mice. These *Cd6*^−/−^ mice (also known as C57BL/6JRj-*Cd6*^em1Ciphe^) were born at expected Mendelian frequencies and lacked detectable CD6 expression (Fig. 5 A).

### Generation of knock-in mice with OST-tagged CD5 molecules

The *Cd5* gene was edited using a double-stranded HDR template (targeting vector) with 5′ and 3′ homology arms of 1,400 bp and 710 bp, respectively. It included an OST coding sequence (Junttila et al., 2005) inserted at the end of the last exon (exon 10) of the *Cd5* gene and a self-excising ACN cassette (Roncagalli et al., 2014) that was introduced at the beginning of the 3′ UTR sequence. The final targeting vector was abutted to a cassette coding for the diphtheria toxin fragment A (Soriano, 1997). The protospacer-adjacent motif present in the targeting vector was destroyed via a silent mutation to prevent CRISPR/Cas9 cleavage. Two sgRNA-specifying oligonucleotide sequences (5′-CAC​CGA​GTG​GCT​CAG​AGA​CTG​TAA​A-3′ and 5′-AAA​CTT​TAC​AGT​CTC​TGA​GCC​ACT​C-3′) were annealed, generating overhangs for ligation into the BbsI site of plasmid pX330 (pSpCas9; Addgene; plasmid ID 42230). JM8.F6 C57BL/6N embryonic stem (ES) cells (Pettitt et al., 2009) were electroporated with 20 µg of targeting vector and 2.5 µg of pX330-sgRNA plasmid. After selection in G418, ES cell clones were screened for proper homologous recombination by Southern blot or PCR analysis. A neomycin-specific probe was used to ensure that adventitious nonhomologous recombination events had not occurred in the selected ES clones. Mutant ES cells were injected into BalbC/N blastocysts. Following germ-line transmission, screening for proper deletion of the ACN cassette and for the presence of the sequence coding for the OST was performed by PCR using the following pair of primers: sense 5′-GAA​GGA​GCC​CTA​CAC​CGA-3′ and antisense 5′-CTA​GGG​GCC​TCT​GTC​CAT-3′. This pair of primers amplified a 354-bp band and a 164-bp band in the case of the *Cd5*^OST^ allele and of the WT *Cd5* allele, respectively. Analysis of mice homozygous for the *Cd5*^OST^ allele showed that their T cells developed properly, yielding normal numbers of mature CD4^+^ and CD8^+^ T cells (Fig. S4 A) that had no defect in proliferation (Fig. S4 B). Mature T cells expressed the CD5 bait at physiological levels (Fig. S4 C) and showed a pattern of TCR-inducible tyrosine phosphorylation comparable to WT T cells (Fig. S4 D). Affinity purified CD5^OST^ bait proteins showed increased phosphorylation after CD3 plus CD4 stimulation, leading to their binding to tyrosine phosphorylated species (Fig. S4 E).

### Design of sgRNA for editing the *Lat* and *Cd6* genes of primary mouse CD4^+^ T cells

Specific sgRNA of high cutting efficiency were designed using the Crispor algorithm and purchased from Synthego. The sequences of the sgRNA used to edit the *Cd6* and *Lat* genes are listed in Table S1. They were modified with 2′-O-methyl 3′ phosphorothioate in the first and last three nucleotides (Hendel et al., 2015). They are intended to introduce a double break located either 4 bp (*Cd6*) or 12 bp (*Lat*) 3′ of the corresponding stop codons.

### Design of DNA HDR templates for editing the *Lat* and *Cd6* genes of primary mouse CD4^+^ T cells

843-bp-long dsDNA HDR templates (Integrated DNA Technologies) were amplified by PCR to edit the *Lat* (Fig. S1) and *Cd6* (Fig. S3) genes. PCR products were purified with the QIAquick PCR purification kit (Qiagen) before being used for nucleofection. To prevent CRISPR/Cas9 cleavage of the edited alleles, a silent mutation destroying the protospacer-adjacent motif sequence present in the genomic DNA was introduced into the dsDNA HDR templates.

### Knock-in of OST-P2A-CD90.1–coding HDR templates, selection, and long-term expansion of appropriately edited CD4^+^ T cells

CD4^+^ T cells were purified (>95% purity) from the spleen and lymph nodes of the C57BL/6 Cas9 mouse (Platt et al., 2014) using the EasySep Mouse CD4^+^ T Cell Isolation Kit (STEMCELL Technologies) or Dynabeads Untouched Mouse CD4^+^ T Cell Kit (Life Technologies), both supplemented with an anti-mouse γδ TCR antibody (clone GL-3; BD Biosciences) to remove γδ T cells. Prior to electroporation, T cells were briefly activated by plating 2.5 × 10^6^ CD4^+^ T cells in a 6-well plate coated with anti-CD3 (clone 2C11; 5 µg/ml) and in the presence of soluble anti-CD28 (clone 37–51; 1 µg/ml), both from Bio X Cell or Exbio. After 48 h of culture at 37°C, T cells were harvested and washed with PBS. A total of 2 × 10^6^ activated T cells were electroporated with a Neon Transfection System (Invitrogen) under the following conditions: voltage (1,500 V), width (20 ms), number of pulses (one), 100 µl tip, and buffer T. Cells were transfected with 5 µg sgRNA and with 10 µg dsDNA HDR template. After electroporation, cells were plated in a 24-well plate with mouse IL (mIL)-2 produced by Concanavalin A stimulation of the DC144 T cell hybridoma (Emilie et al., 1991) and of recombinant mIL-7 (1 ng/ml; Peprotech). Cells were analyzed for the expression of CD90.1 3 d after transfection, and CD90.1^+^ CD4^+^ T cells were sorted on day 5 after transfection using a FACSAria cell sorter (BD Biosciences). After cell sorting, CD90.1^+^ T cells were cultured with IL-2 and IL-7 for 1 d. Cells were then restimulated with plate-bound anti-CD3 (1 µg/ml) and soluble anti-CD28 (1 µg/ml), and the medium was supplemented with IL-2 24 h after restimulation and with IL-2 and IL-7 2 d after restimulation. After 7 d of culture, similar cycles of expansion were repeated until reaching numbers of CD4^+^ T cells appropriate for AP-MS analysis. WT CD4^+^ T cells were grown under the same conditions and used as control in AP-MS experiments.

#### Analysis of the 5′ and 3′ borders of the DNA insertion

Genomic DNA was isolated from the specified CD90.1^+^ CD4^+^ T cells using standard methods (DNeasy Blood & Tissue Kit; Qiagen), and the region straddling the 5′ and 3′ borders of the intended insertion was amplified by PCR using the sets of primers specified in Table S2. The amplified products were sequenced (Eurofins Genomics) following purification (QIAquick PCR Purification Kit; Qiagen). Individual clones were sequenced and compared with the WT sequence.

#### Flow cytometry

Stained cells were analyzed using an LSRII system (BD Biosciences). Data were analyzed with FlowJo V10 (TreeStar). Cell viability was evaluated using SYTOX Blue or DAPI (4′,6-diamidino-2-phenylindole, dihydrochloride; Life Technologies). The following antibodies were used: anti-CD90.1 (HIS51), anti-CD4 (RM4-5), anti-CD8α (53–6.7), anti-CD6 (REA311), anti-TCRβ (H57-597), anti-γδ TCR (GL-3), anti-CD3e (145-2C11), anti-CD44 (IM7), anti-CD25 (PC61), anti-CD45R (RA3-6B2), anti-CD5 (53–7.3), anti-CD62L (MEL-14), and anti-human heparin-binding epidermal growth factor (HB-EGF; BAF259) from BD Biosciences, Miltenyi, R&D Systems, and eBioscience. Note that the human heparin-binding epidermal growth factor constitutes the receptor for diphtheria toxin.

### Mouse CD4^+^ T cell proliferation

T cells were purified by immunomagnetic negative selection using the EasySep Mouse T Cell Isolation Kit and then stimulated with plate-bound anti-CD3 (145-2C11; Exbio) and soluble anti-CD28 (37–51; Exbio) antibodies or with phorbol 12-myristate 13-acetate (PMA) and ionomycin. After 48 h of culture, T cell proliferation was assessed with CellTiter-Glo Luminescent Cell Viability Assay (Promega). The resulting luminescence, which is proportional to the ATP content of the culture, was measured with a Victor 2 luminometer (Wallac; Perkin Elmer Life Science). For CTV-dilution assay, T cells were stained with 5 µM CTV (Molecular Probes) and analyzed by FACS 72 h after stimulation.

### Immunoprecipitation and Western blot analysis of OST-tagged CD4^+^ T cells

The specified OST-tagged CD4^+^ T cells were incubated with anti-CD3 (0.2 µg per 10^6^ cells; 145-2C11) and anti-CD4 (0.2 µg per 10^6^ cells; GK1.5; Exbio) for 15 min on ice, followed by one round of washing at 4°C. Cells were then incubated at 37°C for 5 min and then left unstimulated or stimulated at 37°C by cross-linking for 30 or 120 s with purified goat anti-rat IgG F(ab′)_2_ (0.4 µg per 10^6^ cells; Jackson ImmunoResearch). Stimulation was stopped by the addition of a twice-concentrated lysis buffer (100 mM Tris, pH 7.5, 270 mM NaCl, 1 mM EDTA, 20% glycerol, and 0.4% n-dodecyl-β-D-maltoside) supplemented with protease and phosphatase inhibitors. After 10 min of incubation on ice, cell lysates were centrifuged at 21,000 *g* for 5 min at 4°C. Postnuclear lysates were used for immunoprecipitation or as whole-cell lysates for subsequent immunoblot analysis. For immunoprecipitation, equal amounts of cell lysates were incubated for 1.5 h with specified antibodies. Immune complexes were purified with Pansorbin (Calbiochem) and were washed three times before elution in SDS-containing sample buffer. Eluted samples and whole-cell lysates were loaded on 8% SDS-PAGE gel and subsequently analyzed by immunoblot with specific antibodies. The following antibodies were used for immunoblot analysis: anti-CD5 (SAB4503585; Sigma-Aldrich), anti-CD6 (96123; R&D Systems or H-300; Santa Cruz Biotechnology), anti-LAT (9166; Cell Signaling Technology), anti-phosphotyrosine (4G10; Millipore), anti-UBASH3A (PA5-30637; Thermo Fisher Scientific), anti-VAV1 (2502; Cell Signaling Technology), and anti-GRK6 (5878; Cell Signaling Technology).

### CD4^+^ T cell isolation from CD5^OST^ mice and short-term expansion before AP-MS analysis

CD4^+^ T cells were purified (>95%) from pooled lymph nodes and spleens from CD5^OST^ mice with the Dynabeads Untouched Mouse CD4^+^ T Cell Kit. CD4^+^ T cells were activated with plate-bound anti-CD3 (145-2C11; 5 µg/ml) and soluble anti-CD28 (37–51; 1 µg/ml) antibodies, both from Exbio. After 48 h of culture, CD4^+^ T cells were harvested and grown in the presence of IL-2 (10 U/ml) for 48 h before AP-MS analysis. WT CD4^+^ T cells were subjected to the same expansion protocol and used as controls.

### Stimulation and lysis of short-term– and long-term–expanded mouse CD4^+^ T cells before AP-MS analysis

Short-term–expanded CD4^+^ T cells (100 × 10^6^) from WT and CD5^OST^ mice or long-term–expanded WT, LAT^OST^, and CD6^OST^ CD4^+^ T cells (100 × 10^6^) were incubated with anti-CD3 (0.2 µg per 10^6^ cells; 145-2C11; Exbio) and anti-CD4 (0.2 µg per 10^6^ cells; GK1.5; Exbio) antibodies on ice for 15 min, followed by one round of washing at 4°C. Cells were then incubated at 37°C for 5 min and then stimulated at 37°C with a purified rabbit anti-rat Ig (0.4 µg per 10^6^ cells; Jackson ImmunoResearch) for 30, 120, and 300 s or left unstimulated. Stimulation was stopped by the addition of a twice-concentrated lysis buffer (100 mM Tris, pH 7.5, 270 mM NaCl, 1 mM EDTA, 20% glycerol, and 0.4% n-dodecyl-β-D-maltoside) supplemented with protease and phosphatase inhibitors. After 10 min of incubation on ice, cell lysates were centrifuged at 21,000 *g* for 5 min at 4°C. Postnuclear lysates were then used for affinity purification.

### Affinity purification of OST-tagged protein complexes

Equal amounts of postnuclear lysates were incubated with Strep-Tactin Sepharose beads (IBA GmbH) for 1.5 h at 4°C on a rotary wheel. Beads were then washed five times with 1 ml of lysis buffer in the absence of detergent and of protease and phosphatase inhibitors. Proteins were eluted from the Strep-Tactin Sepharose beads with 2.5 mM D-biotin, a ligand that binds to Strep-Tactin with a higher affinity than the OST sequence does.

### Tandem MS analysis

Following affinity purification, protein samples were air-dried in a Speed-Vac concentrator and either reconstituted in Laemmli buffer and processed by SDS-PAGE and trypsin in-gel digestion as previously described (Voisinne et al., 2019) or reconstituted in 5% SDS–50 mM ammonium bicarbonate and processed for trypsin digestion using an S-trap micro device (Protifi) according to the manufacturer’s instructions. Tryptic peptides were resuspended in 17 µl 2% acetonitrile and 0.05% trifluoroacetic acid and analyzed by nano-LC coupled to tandem MS, using an UltiMate 3000 system (NCS-3500RS Nano/Cap System; Thermo Fisher Scientific) coupled to an Orbitrap Q Exactive MS (model Q Exactive Plus or HFX; Thermo Fisher Scientific). 5 μl of each sample was loaded on a C18 precolumn (300-µm inner diameter × 5 mm; Thermo Fisher Scientific) in a solvent made of 2% acetonitrile and 0.05% trifluoroacetic acid, at a flow rate of 20 µl/min. After 5 min of desalting, the precolumn was switched online with the analytical C18 column (75-µm inner diameter × 50 cm, Acclaim PepMap C18, 2 µM; Thermo Fisher Scientific or in-house packed with 3 µm Reprosil C18) equilibrated in 95% solvent A (5% acetonitrile and 0.2% formic acid) and 5% solvent B (80% acetonitrile and 0.2% formic acid). Peptides were eluted using a 10–45% gradient of solvent B over 60 min at a flow rate of 350 nl/min. The MS was operated in data-dependent acquisition mode with Xcalibur software. On the Q Exactive HFX or Q Exactive Plus MS, MS survey scans were acquired with a resolution of 60,000 or 70,000, respectively, and an AGC target of 3e6. The 12 or 10 most intense ions, respectively, were selected for fragmentation by high-energy collision–induced dissociation, and the resulting fragments were analyzed at a resolution of 15,000 or 17,500, respectively, using an AGC target of 1e5 and a maximum fill time of 22 ms or 50 ms, respectively. Dynamic exclusion was used within 30 s to prevent repetitive selection of the same peptide.

### Protein identification and quantification for interaction proteomics

Raw MS files were processed with MaxQuant software (version 1.5.2.8) for database search with the Andromeda search engine and quantitative analysis. Data were searched against *Mus musculus* entries of the UniProt KB protein database (release UniProtKB/Swiss-Prot+TrEMBL 2017_01, 89,297 entries including isoforms) plus the One-Strep-tag peptide sequence, and the set of common contaminants was provided by MaxQuant. Carbamidomethylation of cysteines was set as a fixed modification, whereas oxidation of methionine, protein N-terminal acetylation, and phosphorylation of serine, threonine, and tyrosine were set as variable modifications. Specificity of trypsin digestion was set for cleavage after K or R, and two missed trypsin cleavage sites were allowed. The precursor mass tolerance was set to 20 ppm for the first search and 4.5 ppm for the main Andromeda database search. The mass tolerance in tandem MS mode was set to 0.5 daltons. Minimum peptide length was set to seven amino acids, and the minimum number of unique or razor peptides was set to one for validation. The I = L option of MaxQuant was enabled to avoid erroneous assignation of undistinguishable peptides belonging to very homologous proteins. Andromeda results were validated by the target decoy approach using a reverse database, with a false discovery rate set at 1% at both peptide sequence match and protein level. For label-free relative quantification of the samples, the match between runs option of MaxQuant was enabled with a match time window of 1 min to allow cross-assignment of MS features detected in the different runs after alignment of the runs with a time window of 20 min. Protein quantification was based on unique and razor peptides. The minimum ratio count was set to 1 for label-free quantification calculation, and computation of the intensity-based absolute quantification (iBAQ) metric was also enabled.

### Data processing and identification of specific interactors

From the proteinGroups.TXT files generated by MaxQuant with the options described above, protein groups with negative identification scores were filtered as well as proteins identified as contaminants. In situations where protein groups corresponded to the same gene name, protein intensities in a given sample were summed over the redundant protein groups. Protein intensities were normalized across all samples by the median intensity. Normalized intensities corresponding to different technical replicates were averaged (geometric mean), and missing values were replaced after estimating background binding from WT intensities. For each bait and each condition of stimulation, we used a two-tailed Welch *t* test to compare normalized log-transformed protein intensities detected in OST-tagged samples across all biological replicates to WT intensities pooled from all conditions of stimulation. To avoid spurious identification of interactors due to missing value imputation, we repeated this process (missing value imputation followed by a two-tailed Welch *t* test) 10 times and estimated fold-changes and P values as their respective average (geometric mean) across all 10 tests. Specific interactors were identified as preys showing a >10-fold enrichment with a P value below 0.005 in at least one condition of stimulation.

### Calculation of interaction stoichiometries

For a given condition of stimulation (represented by the time of stimulation t, with t = 0 s corresponding to the nonstimulated condition), the stoichiometry of the interaction between a prey x and a given bait (denoted bait<x) was computed using

$$S_{bait<x}(t)\;=\;<I_{OST,x}(t)>/<I_{OST,bait}(t)>*N_{pep,bait}/N_{pep,x}.$$

<I_OST,x_> corresponds to the normalized intensity of protein x in OST-tagged samples and stimulation time t averaged (geometric mean) across all biological replicates, and N_pep_ corresponds to the number of tryptic peptides, theoretically observables as estimated from iBAQ values. We also computed stoichiometries independently for each biological replicate and used these values to quantify the regulation of bait–prey association following TCR engagement. For a given condition of stimulation, log-transformed stoichiometries were compared with that of the nonstimulated condition using a two-tailed Welch *t* test. We selected preys whose interaction stoichiometry changed at least twofold with a P value below 0.05 in at least one condition of stimulation compared with the nonstimulated condition.

### High-resolution MS characterization of long-term–expanded CD4^+^ T cell proteome

For proteome analysis, cell pellets corresponding to 5 × 10^6^ long-term–expanded CD4^+^ T cells were incubated with 150 µl of lysis buffer containing 50 mM Tris, pH 7.5, 0.5 mM EDTA, 135 mM NaCl, and 1% SDS for 10 min on ice and subjected to sonication with a Bioruptor ultrasonicator. Protein concentration was determined using a detergent-compatible assay (DC assay; Bio-Rad), and an aliquot of 100 µg of each sample was migrated briefly on SDS–PAGE gel and processed for in-gel digestion as previously described (Voisinne et al., 2019). Tryptic peptides were resuspended in 125 µl of 2% acetonitrile and 0.05% trifluoroacetic acid. 5 µl of each sample was analyzed with nanoLC-MS on the Q Exactive HFX as described above, but with a longer separation gradient (10–45% of solvent B over 120 min). Raw MS files were processed with MaxQuant as described above, except that phosphorylation was not included as a variable modification for the database search.

### Calculation of copy numbers per T cell and of fraction of cell mass occupied by a protein

Analysis of the proteome of long-term–expanded CD4^+^ T cells identified 6,261 protein groups. Protein entries from the MaxQuant proteinGroups.TXT output were first filtered to eliminate entries from reverse and contaminant databases. Cellular protein abundances were determined from raw intensities using the protein ruler methodology (Wiśniewski et al., 2014), using the following relationship: protein copies per cell = (protein MS signal × N_A_ × DNA mass)/(M × histone MS signal), where N_A_ is Avogadro’s constant, M is the molar mass of the protein, and the DNA mass of a diploid mouse cell is estimated to be 5.5209 pg. Cellular protein abundances were averaged (geometric mean) over biological replicates. Overall, the cellular protein abundance could be estimated for 5,773 protein groups. The cellular protein abundance of short-term–expanded WT CD4^+^ T cells is from Voisinne et al. (2019). For long- and short-term proteomes, the fraction of cell mass occupied by a protein was calculated from protein abundance (N) and molecular weight (MW) using the formula: protein (j) mass fraction = N_j_*MW_j_/sum_i_(N_i_*MW_i_), where the sum runs over all protein groups.

### Bulk RNA-sequencing and analysis

Naive CD4^+^ T cells were purified (purity >95%) from pooled lymph nodes and spleens from WT, *Cd5*^−/−^, *Cd6*^−/−^, and *Cd5*^−/−^*Cd6*^−/−^ mice using the EasySep Mouse Naive CD4 T^+^ cell Isolation Kit. WT (human DTR^+^) and *Lat*^−/−^ (human DTR^−^) naive CD4^+^ T cells were purified from pooled lymph nodes and spleens from *Lat*^fl-dtr^ maT-Cre mice using FACS AriaIII as described (Roncagalli et al., 2014). The various purified naive CD4^+^ T cells were stimulated with plate-bound anti-CD3 (145-2C11; 3 µg/ml) and soluble anti-CD28 (37–51; 1 µg/ml) antibodies for 20 h or left unstimulated. CD25, CD44, and CD69 were up-regulated and CD62L down-regulated on all the anti-CD3 plus anti-CD28–treated T cells, which demonstrated that they had been evenly stimulated. RNA was isolated using an RNeasy Plus mini kit (Qiagen). All samples were analyzed on an Agilent 2100 Bioanalyzer and passed quality control based on the RNA Integrity Number (≥8). DNA libraries were constructed by GenomEast platform using TruSeq Stranded mRNA Library Prep (Illumina). The DNA libraries were subjected to high-throughput sequencing on the Illumina Hiseq 4000 as single-read 50-base reads following Illumina’s instructions. The fastq files were assessed with the fastqc program, and trimming was performed with DimerRemover to remove Illumina adapters and low-quality reads. Reads were mapped to the mouse GRCm38 (mm10) reference genome using STAR, and the number of reads mapped to each gene was determined with featureCounts v1.6.0. Raw read counts were combined into a numeric matrix, with genes in rows and experiments in columns, and used as input for differential gene expression analysis with DESeq2 v1.22.2 after removing genes with less than one total read across all samples. Normalization factors were computed on the filtered data matrix using the concept of variance stabilizing transformations. Pairwise comparisons were performed between treatment groups to obtain log_2_ fold change, and adjusted P values were corrected for multiple testing using the Benjamini-Hochberg (BH) method and used to select genes with significant expression differences (q < 0.05).

### Deletion of the CD5 and CD6 genes in primary CD4^+^ T cells

CD4^+^ T cells were purified by immunomagnetic negative selection from mice constitutively expressing Cas9 and edited as described above. The CD5 or CD6 genes were ablated using the sgRNA specified in Table S2. The efficiency of gene deletion was checked by flow cytometry on day 3 after transfection. Edited cells were kept in culture in the presence of IL-2 (10 U/ml) and IL-7 (1 ng/ml) for 7 d after nucleofection. Cells were then labeled with 5 µM CTV (Molecular Probes), restimulated with anti-CD3 and anti-CD28 antibodies (1 µg/ml), and analyzed 48 h later by FACS for CTV content.

### Data availability

The MS proteomics data have been deposited in the ProteomeXchange Consortium via the PRIDE partner repository (http://www.ebi.ac.uk/pride) with the dataset identifiers: PXD018526 (LAT interactome of long-term–expanded CD4^+^ T cells), PXD018527 (CD6 interactome of long-term–expanded CD4^+^ T cells), PXD018552 (CD5 interactome of short-term–expanded CD4^+^ T cells), and PXD018766 (proteome of long-term–expanded CD4^+^ T cells). RNA-sequencing data have been deposited in the Gene Expression Omnibus public database under accession no. GSE148721.

### Online supplemental material

Fig. S1 shows the structure of the HDR template used to edit the *Lat* gene and sequences of the resulting junctions. Fig. S2 shows a comparison of the fraction of cell mass occupied by the proteins quantified in both short-term– and long-term–expanded CD4^+^ T cells. Fig. S3 shows mouse primary CD4^+^ T cells amenable to fast-track AP-MS characterization of the CD6 signalosome. Fig. S4 shows the normal development and function of T cells of CD5^OST^ mice. Fig. S5 shows augmented TCR-mediated activation in primary WT CD4^+^ T cells rendered CD5 deficient. Table S1 shows the sgRNA sequences. Table S2 shows the primer sequences. Data S1 compares the proteomes of long-term– and short-term–expanded CD4^+^ T cells. Data S2 lists the bait–prey interactions identified in the CD6, CD5, and LAT interactomes. Data S3 lists differentially expressed genes in CD4^+^ T cells of the specified genotype activated for 20 h with anti-CD3 plus anti-CD28 antibodies versus their unstimulated counterparts.

## Supplementary Material

Table S1 shows the sgRNA sequences.

Table S2 shows the primer sequences.

Data S1 compares the proteomes of long-term– and short-term–expanded CD4^+^ T cells and the proteins identified in long-term– and short-term–expanded CD4^+^ T cells along with their cellular abundance (number of copies per cell) and the fraction of cell mass they occupy.

Data S2 lists the bait–prey interactions identified in the CD6 and LAT interactomes of CRISPR/Cas9–edited and long-term–expanded primary CD4^+^ T cells and in the CD5 interactome of CD4^+^ T cells that were isolated from CD5^OST^ mice and underwent short-term expansion.

Data S3 lists differentially expressed genes in CD4^+^ T cells of the specified genotype activated for 20 h with anti-CD3 plus anti-CD28 antibodies versus their unstimulated counterparts.
