# Supplementary material for: The T cell CD6 receptor operates a multitask signalosome with opposite functions in T cell activation
Source: J Exp Med. 2020 Oct 30;218(2):e20201011. doi: 10.1084/jem.20201011 (PMC7608068; doi:10.1084/jem.20201011)
Supplement: Table S1 — shows the sgRNA sequences. [file JEM_20201011_TableS1.docx]

Table S1. sgRNA sequences

| Target | Name | Sequence |
| --- | --- | --- |
| CD6 (OST) | 116R | 5′-GAGCCTCGGGGACCCAGGCT-3′ |
| LAT | 81F | 5′-CACCTCTTCCTGGAAAGCTC-3′ |
| CD5 (KO) | CD5-1 | 5′-CAGCACTTCGTGGGAGTCCA-3′ |
| CD5 (KO) | CD5-2 | 5′-CACATACCTGCTGGGAACGC-3′ |
| CD6 (KO) | CD6-1 | 5′-GGAGACGGTTGTGCCACCGA-3′ |
| CD6 (KO) | CD6-2 | 5′-CCAAGGAAGAGCCACATGTC-3′ |
| EGFP | sgEGFP | 5′−GGGCGAGGAGCTGTTCACCG-3′ |
