# Supplementary material for: The T cell CD6 receptor operates a multitask signalosome with opposite functions in T cell activation
Source: J Exp Med. 2020 Oct 30;218(2):e20201011. doi: 10.1084/jem.20201011 (PMC7608068; doi:10.1084/jem.20201011)
Supplement: Table S2 — shows the primer sequences. [file JEM_20201011_TableS2.docx]

Table S2. Primer sequences

| Cd6-1 | 5′-AATAACTAGCTCGGGTCTTCCGCAGGG-3′ |
| --- | --- |
| Cd6-2 | 5′-GTGGACTCAGAGACCTCTGGAAGCAAGC-3′ |
| Cd6-3 | 5′-CCCTACTGACCTGATTCTCTTGTCCCCC-3′ |
| Cd6-4 | 5′-GAGGATGAGGGGCTGGGAGAATGCCCAC-3′ |
| Lat-1 | 5′-AGAGGCGTCTCTGGGTAGGTGACTCTG-3′ |
| Lat-2 | 5′-CCTTTATTCCATGACATGGTCTGGCTGG-3′ |
| Lat-3 | 5′-CTCAGCCTCTGTGAACTCCCAGGAGGTG-3′ |
| Lat-4 | 5′-CACAAAGAATGTGCCTAACCACTGGGG-3′ |
| Cd90.1-1 | 5′-TGAAGTTGGCTAGGGTAAGGACCTTGAT-3′ |
| Cd90.1-2 | 5′-CCTGGACTGCCGCCATGAGAATAACACC-3′ |
